# Clinical and genetic analyses of a Swedish patient series diagnosed with ataxia

**DOI:** 10.1007/s00415-023-11990-x

**Published:** 2023-10-03

**Authors:** Sorina Gorcenco, Efthymia Kafantari, Joel Wallenius, Christin Karremo, Erik Alinder, Sigurd Dobloug, Maria Landqvist Waldö, Elisabet Englund, Hans Ehrencrona, Klas Wictorin, Kristina Karrman, Andreas Puschmann

**Affiliations:** 1 Neurology, Department of Clinical Sciences Lund, Lund University, Skåne University Hospital, Lund, Sweden; 2 Division of Clinical Sciences Helsingborg, Department of Clinical Sciences Lund, Faculty of Medicine, Lund University, Lund, Sweden; 3 Pathology, Department of Clinical Sciences Lund, Lund University, Skåne University Hospital, Lund, Sweden; 4 Division of Clinical Genetics, Department of Laboratory Medicine, Lund University, Lund, Sweden; 5 Department of Clinical Genetics, Pathology and Molecular Diagnostics, Office for Medical Services, Region Skåne, Lund, Sweden; 6 SciLifeLab National Research Infrastructure, Solna, Sweden

**Keywords:** Hereditary ataxia, Next-generation sequencing, Post-NGS phenotyping

## Abstract

Hereditary ataxia is a heterogeneous group of complex neurological disorders. Next-generation sequencing methods have become a great help in clinical diagnostics, but it may remain challenging to determine if a genetic variant is the cause of the patient’s disease. We compiled a consecutive single-center series of 87 patients from 76 families with progressive ataxia of known or unknown etiology. We investigated them clinically and genetically using whole exome or whole genome sequencing. Test methods were selected depending on family history, clinical phenotype, and availability. Genetic results were interpreted based on the American College of Medical Genetics criteria. For high-suspicion variants of uncertain significance, renewed bioinformatical and clinical evaluation was performed to assess the level of pathogenicity. Thirty (39.5%) of the 76 families had received a genetic diagnosis at the end of our study. We present the predominant etiologies of hereditary ataxia in a Swedish patient series. In two families, we established a clinical diagnosis, although the genetic variant was classified as “of uncertain significance” only, and in an additional three families, results are pending. We found a pathogenic variant in one family, but we suspect that it does not explain the complete clinical picture. We conclude that correctly interpreting genetic variants in complex neurogenetic diseases requires genetics and clinical expertise. The neurologist’s careful phenotyping remains essential to confirm or reject a diagnosis, also by reassessing clinical findings after a candidate genetic variant is suggested. Collaboration between neurology and clinical genetics and combining clinical and research approaches optimizes diagnostic yield.

**Supplementary Information:**

The online version contains supplementary material available at 10.1007/s00415-023-11990-x.

## Introduction

Ataxia is a neurological sign with incoordination of movements resulting from dysfunction of the cerebellum and its afferent and efferent pathways. According to the location of the underlying dysfunction, ataxias can be classified as cerebellar, sensory, and vestibular. The main manifestations of ataxia are gait impairment, limb incoordination, nystagmus, and slurred speech. Ataxia can be subdivided into sporadic, hereditary, and acquired forms [1, 2]. Monogenetic ataxias are chronically progressive neurological disorders that can be further categorized by their inheritance pattern and underlying genetic causes into autosomal dominant cerebellar ataxias (ADCAs), autosomal recessive cerebellar ataxias (ARCAs), X-linked cerebellar, and mitochondrial ataxias. The hereditary ataxias are a large and heterogeneous group of diseases with variable genetic, clinical, pathogenic, pathophysiologic, and neuropathologic features [3].

Massively parallel sequencing, also called Next-generation sequencing (NGS), is increasingly being used for clinical diagnostics and greatly facilitates the identification of the underlying genetic causes. However, the diagnostic yield of NGS for ataxia is only between 12 and 52% [4], and approximately 30% of patients with clinical suspicion of ADCAs and 50% of ARCAs remain undiagnosed [5–7]. In recent years, the technical methodology to identify various types of genetic variants through NGS has dramatically increased, but it can remain very challenging to firmly decide if a detected genetic variant indeed is the cause of the examined patient’s or family’s disorder. In 2015, the American College of Medical Genetics and Genomics (ACMG) developed guidelines for the interpretation of sequence variants that have become quasi-universally accepted and applied. However, it has also been pointed out that they may be suboptimal for diagnosing very rare disorders such as ataxias [8], especially when they are used in a standard clinical setting where only those results of genetic analyses that are considered highly likely disease causes are sent to the neurologists [9].

Here, we present a cohort of patients with ataxia of known or unknown etiology from southern Sweden. These patients and many of their family members have been studied clinically and radiologically, and we investigated the molecular etiology of previously undiagnosed cases. In our study, we used a collaborative approach where possible genetic disease causes were revealed to the treating neurologists and discussed between neurologists, bioinformaticians as well as medical and clinical geneticists.

## Methods

### Recruitment and selection of patients

Patients were identified through a search for the diagnosis of hereditary ataxia (ICD-10 Version: 2019 (who.int) G11.0, G11.1, G11.2, G11.3, G11.8 or G11.9) between the years of 2011–2020 in the diagnosis register of the department of neurology at Skåne University Hospital. We also recruited patients with the diagnosis of hereditary ataxia who were referred by other neurologists, from contact with the Swedish patient organization SCA-Network, or through their families. Patients with SCA3 were specifically targeted for a multicenter study. Patients with Friedreich ataxia from our center were already included in a previous study [10] and these were not approached again. A research nurse sent a letter with detailed information about the project, written by the research group, and a request to send back a response form (interested/not interested) by mail to each patient found to have a clinical diagnosis of hereditary ataxia. A more detailed description of patient recruitment in this study has recently been published [11].

### Clinical examination

All the included patients were seen by a study doctor and a study nurse during a research visit at our clinic, or during home visits. All were interviewed following a standardized checklist for medical and family history. Results from brain imaging, nerve conduction studies, analysis of cerebral spinal fluid, and genetic examination (if available) were retrieved from the clinical records. The neurological examination was conducted using a standardized protocol for the examination of patients with ataxia, focusing on speech, eye movements, coordination of movements, and gait. To assess the disease severity, we used the Scale for the Assessment and Rating of Ataxia (SARA) [12]. Family pedigrees were drawn based on probands’ and relatives’ information.

Radiology reports and when available, original images, were reviewed by the authors for cerebellar and spinal cord atrophy. Records from nerve conduction studies, if existing, were evaluated for peripheral nerve impairment. Blood samples were collected by our research nurse from each patient after the clinical examination. In addition, cerebral spinal fluid was collected by the main author from patients with spinocerebellar ataxia type 3 (SCA3) for a multicenter study [13, 14].

### Genetic analyses

Some of the patients recruited here underwent genetic analyses as part of their clinical workup, others were analyzed within this study:

- Prior to this study: some patients had already an established genetic diagnosis. However, blood samples were collected for storage in the biobank for future analyses. Many patients had been analyzed for repeat expansions causing SCA1, 2, 3, 6 and 7 in a test package that was widely used at our hospital; others had been examined for particular genes based on their phenotype. More recently, a number of patients had undergone gene panel analyses based on targeted resequencing, Whole Exome Sequencing (WES) or Whole Genome Sequencing (WGS).
- Within this study: the patients without a genetic diagnosis were tested using WES or WGS methods. The choice between WES and WGS was made based on the clinical presentation, family history, and availability at the time of testing. Study bioinformaticians also re-analyzed available raw data from clinical WES analyses from three patients. Most WES or WGS analyses were performed at the Center for Translational Genomics at Lund University. Additionally, WES, WGS, and genotyping of family members was performed by Centogene, Rostock, Germany, or BluePrint Genetics, Helsinki, Finland.

### Bioinformatic analyses

WES and WGS data were analyzed for single nucleotide variants and short insertions or deletions, repeat expansions (short tandem repeats), and copy number variants (deletions, duplications). Variant Call Format (VCF) files were annotated with Variant Effect Predictor (VEP).

*Single nucleotide variants* (SNVs) and short insertions and deletions were prioritized based on their CADD score [15]. SNVs were annotated further with the dbNSFP plugin, while synonymous and intronic variants were evaluated with appropriate freely available software (Trap score [16], PredictSNP2 [17]). Splice region variants were interpreted with spliceAI [18]. Vt tool was used for calls’ decomposition [19]. Only rare variants (MAF < 0.01) in gnomAD non-Finnish Europeans, 1000 Genomes, and SweGen [20] frequency databases and with genotype quality higher than 20 were selected. Variants in genes present in an in-house gene list containing 1020 related ataxia genes compiled from the Human Phenotype Ontology (HPO) entry on ataxia (HP:0001251, accessed in 2021) and ataxia gene panels were assessed further. The variant classification was performed based on the guidelines published by the American College of Medical Genetics and Genomics (ACMG) in 2015 [21], and we only considered candidate variants those that were classified as pathogenic, likely pathogenic or of uncertain significance (VUS) by the classifiers used (Varsome [22], Franklin by Genoox Franklin (genoox.com)). We also searched the variants in ClinVar database.

*Expanded short tandem repeats* were detected with ExpansionHunter 5.0.0, using the bundled hg19 short tandem repeat catalog and default settings. A total of 44 known short tandem repeats that may be implied in ataxia were selected from ExpansionHunter 5.0.0 and the literature [23], and examined. The outputs of ExpansionHunter were then visualized with REViewer 0.2.7, facilitating manual curation of the reads spanning the short tandem repeat. This manual curation followed the tutorial and guidelines available at https://www.illumina.com/science/genomics-research/articles/reviewer-alignments-short-reads-long-repeat.html and REViewer/docs at master · Illumina/REViewer · GitHub.

*Copy number variants* (CNVs) of the WES data were searched using ExomeDepth [24]. ExomeDepth is an R package with which CNVs can be detected from targeted sequence data, typically exome sequencing.

A different software is required to search for CNVs in WGS samples. GATK was chosen for this purpose, run in 'cohort' mode with otherwise default settings as described in the official guide available here: https://gatk.broadinstitute.org/hc/en-us/articles/360035531152--How-to-Call-common-and-rare-germline-copy-number-variants. CNVs were analyzed in a manually curated in-house gene list of 320 genes, compiled from gene lists used for clinical diagnostics at the Dept. of Clinical genetics in Lund and commercially available ataxia gene panels. The gene list can be made available by the authors upon request.

### “Post-NGS phenotyping”

Very rare variants in ataxia-related genes that were classified as pathogenic, likely pathogenic, or of uncertain significance with a high likelihood of pathogenicity were discussed in rounds of conferences with clinicians, bioinformaticians, and a medical geneticist. These results were then verified using post-NGS phenotyping. The first author reevaluated all new genetic findings in relationship to the presenting clinical phenotype, genetic databases, and reported cases in the literature. When the findings did match (see criteria below), orthogonal validation testing was performed if necessary and feasible.

The following criteria made us consider a variant to be compatible with the patient’s/family’s phenotype:

- Family history is consistent with the mode of inheritance of the disorder
- The patient and the affected family members had a well-defined syndrome; we were looking for a specific signature of neurological and/or non-neurological disease phenotypes and compared between the patient and previous publications about the particular disorder, and, in the case of families, between affected individuals of a family [21]
- Careful re-appreciation of the genetic results and database findings (variant frequency in the population, prediction tools, genotype, quality of sequencing, validity of bioinformatic methods, etc.).

In several instances, if patients described other family members with similar symptoms, these family members were invited to participate in our study. We also tested unaffected family members for analyses of co-segregation of disease with genotype or to determine if two variants in the same gene in a proband were in *cis* or in *trans*.

## Results

As described previously, 158 ataxia patients were identified and contacted [11]. In the present study, 87 patients with the diagnosis of hereditary ataxia were included. From 91 patients who had been examined within the study, four were excluded because of other diagnoses that were found after clinical examination and reevaluation of each patient’s neurological records: one patient was diagnosed with multiple system atrophy type C, one with an adult form of spinal muscular atrophy, one with functional dystonia and one with a paramalignant syndrome. Patient demographic data is presented in Table 1.

**Table 1:** Demographics of the hereditary ataxia study group

	Total sample (*n* = 87)		Male (*n* = 49)		Female (*n* = 38)		With genetic diagnosis (*n* = 44)		Without genetic diagnosis (*n* = 43)	
	*M*	SD	*M*	SD	*M*	SD	*M*	SD	*M*	SD
Age (years)	55.9	16.2	57.9	17.1	53.3	14.8	56.6	15.0	55.2	17.5
Age at disease onset (years)	38.7	19.1	40.8	19.1	36.0	19.1	36.3	18.7	41.3	19.5
Disease duration (years)	17.0	12.1	16.7	11.6	17.5	12.7	19.7	12.7	14.4	11.0
SARA score (points)	13.0	9.1	12.5	8.1	13.6	10.3	13.5	8.8	12.5	9.3

This table shows the mean values and standard deviations for age, age at disease onset, disease duration, and SARA score for the total sample of patients and separately for the group of male patients, female patients, patients with a genetic diagnosis and patients without a genetic diagnosis. No statistically significant difference was seen between the mean values of male and female patients when comparing the means with an independent sample *t* test (*p* > 0.05). However, when comparing the groups of patients with or without a genetic diagnosis a significant difference was seen for disease duration, the patients with a genetic diagnosis have had an approximately 5 years longer disease duration compared with the patients without a diagnosis (*p* < 0.05)

*M* mean, *n* total number, *SARA* scale for the assessment and rating of ataxia, *SD* standard deviation

Twenty-seven patients had a genetic diagnosis before inclusion and two of their relatives were found to be presymptomatic carriers; together they came from 19 families (Online Resource 1). Fifteen patients, from 11 families, received a confirmed genetic diagnosis within our study (Table 2). At the end of this study, 44 (50.6%) of 87 individuals from 30 (39.5%) of 76 families had a confirmed genetic diagnosis. In an additional 8 probands, we remained uncertain if the variants in ataxia genes identified can explain the patient’s phenotype; we encountered different diagnostic situations as outlined in Online Resource 2.

**Table 2:** Study patients who received a genetic diagnosis within research analyses

Proband only/Family	Patient ID	Cerebellar signs/Pyramidal signs/Other clinical details	Gene(s), Transcript(s), Variant(s), Genotype, Test method(s)	Database information (Manual variant classification by Clinical geneticist)	Genetic diagnosis (Comment)
Proband only	P1011	AO 15 years. SD 39 years. Gait ataxia. SARA 4. SARA/SD = 0.10/y. GEN, hypometric saccades, ocular apraxia, square wave jerks on fixation. MRI/CT: severe CA Clonus, LL hyperreflexia Decreased night vision. Anxiety	*SAMD9L* NM_152703.2 c.2956C > T p.(Arg986Cys) het WES (see Comment)	ClinVar: Pathogenic (ID 446530) gnomAD NFE genomes & Swefreq: Absent gnomAD NFE exomes: 0,000016 CADD-phred: 24.5 Varsome ACMG: Pathogenic Franklin by Genoox: Likely pathogenic	Ataxia pancytopenia syndrome Variant was not automatically detected in peripheral blood but was present there in a low percentage (mosaicism) upon renewed examination. It was detected by genotyping DNA from a buccal swab within a research study. The patient's brother had hematological disease and the *SAMD9L* variant was first detected in that brother This patient and family were reported in [50] and [25]
Family P1017_ P1018_ P1027 (Fig. 1A)	P1017 (proband)	AO 42 years. SD 7 years. Mild gait & UL ataxia. Dysarthria. SARA 11. SARA/SD = 1.57/y. Horizontal nystagmus. MRI/CT: moderate CA No pyramidal signs Mild cognitive impairment, mild macrognathia, attached earlobes, decreased gaze fixation & decreased night vision, dysphagia	*SAMD9L* NM_152703.2 c.2640C > A p.(His880Gln) het WES (peripheral blood)	ClinVar: Pathogenic—Likely pathogenic (ID 242372) gnomAD NFE genomes & exomes; Swefreq: Absent CADD-phred: 17 Varsome ACMG: VUS Franklin by Genoox: VUS Manual ACMG: Likely pathogenic (PM2 + PS4 + PP1); AD condition	Ataxia pancytopenia syndrome
	P1018 (proband’s daughter)	AO 14 years. SD 5 years. Mild gait ataxia. SARA 1. SARA/SD = 0.2/y. Vertical nystagmus. MRI/CT: mild CA & brainstem atrophy, mild white matter lesions No pyramidal signs History of cytopenia. Sensorimotor neuropathy, macrognathia			Ataxia pancytopenia syndrome
	P1027 (proband’s brother)	AO 18 years. SD 28 years. Mild gait ataxia. SARA 3. SARA/SD = 0.11/y. Downbeat vertical nystagmus. MRI/CT: severe CA & brainstem atrophy No pyramidal signs Orthostatic hypotension			Ataxia pancytopenia syndrome
Family P1073_ P1091_ P1093 (Fig. 1B)	P1073 (proband)	AO 41 years. SD 30 years. Gait, UL & LL ataxia. SARA 8. SARA/SD = 0.27/y. Horizontal & vertical nystagmus, hypometric saccades, saccadic pursuit, high gain VOR. MRI/CT: moderate CA No pyramidal signs Hyporeflexia, impaired vibration sense, rigidity, strabismus, unilateral ptosis, diplopia. Psoriasis	*ELOVL4* NM_022726.4 c.511A > C p.(Ile171Leu) het WGS/WES	ClinVar: NR gnomAD NFE genomes & exomes; Swefreq: Absent CADD-phred: 28.9 Varsome ACMG: VUS Franklin by Genoox: VUS Manual ACMG: VUS – > Likely pathogenic after segregation analysis * P1092’s daughter P1097 was examined outside this study. She showed typical symptoms (see text) and carried the same variant	SCA34. See text; ACMG classification was changed due to our study
	P1091 (proband’s brother)	AO 50 years. SD 18 years. Gait, UL & LL ataxia. SARA 7.5. SARA/SD = 0.42/y. GEN, low gain & saccadic smooth pursuit, hypometric saccades. MRI/CT: severe CA No pyramidal signs Areflexia, epileptic seizures, depression			SCA34. See text; ACMG classification was changed due to our study
	P1092 (proband’s cousin)	AO 63 years. SD 10 years. Gait, UL & LL ataxia. SARA 6.5. SARA/SD = 0.65/y. Hypometric saccades, saccadic & low gain pursuit. MRI/CT: severe CA No pyramidal signs impaired vibration sense, epileptic seizures, psoriasis, constipation, cognitive impairment, insomnia			SCA34. See text; ACMG classification was changed due to our study
Proband only	P1037	AO 4 years. SD 28 years. Gait, UL & LL ataxia. Dysarthria. SARA 11. SARA/SD = 0.39/y. MRI/CT: normal No pyramidal signs Sensorimotor neuropathy, hearing loss, vision loss	*SLC52A2* NM_024531.4 c.968 T > C p.(Leu323Pro) hom Targeted panel [26]	ClinVar: Likely pathogenic (ID 979035) gnomAD NFE genomes & Swefreq: Absent gnomAD NFE exomes: 0,0000329 CADD-phred: 26.3 Varsome ACMG: VUS Franklin by Genoox: Likely pathogenic	Brown–Vialetto–Van Laere syndrome-2 Previously published: [26]
Proband only (Fig. 1C)	P1040	AO 63 years. SD 18 years. Gait, UL & LL ataxia. Dysarthria. SARA ND. Hypermetric saccades. MRI/CT: severe CA & hippocampus atrophy. Moderate cerebral atrophy No pyramidal signs Motor restlessness, disinhibition, perseveration Post mortem: Typical/pathognomonic neuropathology	*STUB1* NM_005861.2 c.107 T > C p.(Leu36Pro) het WES	ClinVar: NR gnomAD NFE genomes & exomes; Swefreq: Absent CADD-phred: 24.8 Varsome ACMG: VUS Franklin by Genoox: VUS Manual ACMG before neuropathology: VUS (PM2 + PP5)	Spinocerebellar ataxia 48
Proband only (Fig. 1D)	P1002	AO 18 years. SD 23 years. Gait (w), UL & LL ataxia. Dysarthria. SARA 37. SARA/SD = 1.61/y. Ocular motor apraxia, hypometric saccades, omnidirectional ophthalmoplegia MRI/CT: moderate CA & cerebral atrophy Spasticity Cognitive impairment, hearing loss, dysphagia, UL + LL muscle atrophy, urinary incontinence, orthostatic hypotension Consanguineous parents	*STUB1* NM_005861.2 c.761G > A p.(Arg254His) hom WES	ClinVar: NR gnomAD NFE genomes: 0,0000321 gnomAD NFE exomes & Swefreq: Absent CADD-phred: 28.3 Varsome ACMG: Pathogenic Franklin by Genoox: Likely pathogenic Manual ACMG: VUS (PM2 + PM5 + PP5)	Autosomal recessive spinocerebellar ataxia 16
Proband only (Fig. 1E)	P1058	AO 37 years. SD 14 years. Gait (w)& LL ataxia. SARA 13. SARA/SD = 0.93/y. Hypometric saccades. MRI/CT: normal Hyperreflexia Tremor at rest, rigidity, bradykinesia, muscle cramps in calves and thighs, depression, myopia	*SPAST* NM_014946.4 c.722del p.(His241Profs*13) het WES	ClinVar: NR gnomAD NFE genomes & exomes; Swefreq: Absent CADD-phred: 29.7 Varsome ACMG: Likely pathogenic Franklin by Genoox: Likely pathogenic Manual ACMG: Likely pathogenic (PVS1 + PM2). AD condition	Autosomal dominant spastic paraplegia 4
Proband only (Fig. 1F)	P1048	AO 27 years. SD 9 years. Gait & LL ataxia. SARA 2. SARA/SD = 0.22/y. MRI/CT: normal Hyperreflexia, EPR, clonus Bradykinesia	*CAPN1* NM_005186.4 c.759 + 1G > A splice donor variant hom WES	ClinVar: Pathogenic gnomAD NFE genomes & exomes; Swefreq: Absent CADD-phred: 34 Varsome ACMG: Pathogenic Franklin by Genoox: Pathogenic Manual ACMG: Pathogenic (PVS1 + PM2 + PP5). AR condition	Autosomal recessive spastic paraplegia 76
Proband only (Fig. 1G)	P1070	AO 55 years. SD 18 years. Gait, UL & LL ataxia. Dysarthria. SARA 15.5. SARA/SD = 0.86/y. GEN, downbeat nystagmus, saccadic smooth pursuit, slow saccades, hypoactive VOR. MRI/CT: mild CA No pyramidal signs Areflexia, sensory neuropathy, impaired proprioception and vibration sense, paresthesia, urinary dysfunction, orthostatic hypotension; bradykinesia, UL dystonia, tremor, depression	*RFC1* NM_002913.5 AAGGG (normal: AAAAG) Repeat lengths were not reported. See Online Resource 4. Biallelic WGS Confirmed by standard flanking PCR and repeat prime PCR at Genetic Services Laboratory, The University of Chicago, USA. Repeat lengths were not reported	ClinVar: NR gnomAD NFE genomes & exomes; Swefreq: NR CADD-phred: NR	Cerebellar ataxia, neuropathy, and vestibular areflexia syndrome (Sister had ataxia since age 55 and unexplained cough for many years; genetic testing of the sister is currently pending.)
Proband only (Fig. 1H)	P1095	AO 60 years. SD 12 years. Gait, UL & LL ataxia. SARA 8.5. SARA/SD = 0.04/y. GEN, hypoactive VOR. MRI/CT: normal No pyramidal signs Sensory polyneuropathy, impaired proprioception, impaired vibration sense, areflexia, muscle cramps in lower extremities, dizziness, depression	*RFC1* NM_002913.5 AAGGG (normal: AAAAG) Repeat lengths estimated to be 29 and 38 but difficult to measure with certainty. See Online Resource 4. Biallelic WGS Confirmed by standard flanking PCR and repeat prime PCR at Genetic Services Laboratory, The University of Chicago, USA. Repeat lengths were not reported	ClinVar: NR gnomAD NFE genomes & exomes; Swefreq: NR CADD-phred: NR	Cerebellar ataxia, neuropathy, and vestibular areflexia syndrome Confirmed by standard flanking PCR and repeat prime PCR at Genetic Services Laboratory, The University of Chicago, USA. Repeat lengths were not reported
Proband only (Fig. 1I)	P1089	AO 57 years. SD 7 years. UL & LL ataxia. SARA 3.5. SARA/SD = 0.50/y. MRI/CT: mild medial temporal atrophy No pyramidal signs Irritability, cognitive impairment, insomnia, urinary dysfunction, constipation, decreased sensibility for pain & temperature, impaired vibration sense, tremor	*HTT* NM_002111 36 repeats (CAG only) Monoallelic See Online Resource 4 WGS Confirmed by standard flanking PCR and repeat prime PCR at Centogene, Germany. Repeat lengths with that method determined as 36 ± 1 (and 15 ± 1)	ClinVar: Pathogenic with reduced penetrance gnomAD NFE genomes & exomes; Swefreq: NR CADD-phred: NR	Huntington’s disease with expanded allele with reduced penetrance

Clinical phenotype and genetic findings of patients that were analyzed within the research

*ACMG* American College of Medical Genetics, *AD* autosomal dominant, *AO* age at onset, *AR* autosomal recessive, *CA* cerebellar atrophy, *CADD-phred* combined annotation dependent depletion, a tool for scoring the deleteriousness of single nucleotide variants as well as insertion/deletions variants in the human genome, *comp het* compound heterozygosity, *CT* computed tomography, *EPR* extensor plantar response, *GEN* gaze-evoked nystagmus, *het* heterozygosity, *hom* homozygosity, *LL* lower limbs, *MRI* magnetic resonance imaging, *ND* not determined, *NFE* Non-Finnish-European, *NR* not reported, *SARA* scale for the assessment and rating of ataxia, *SD* symptom duration (time from symptom onset to examination within the study), *UL* upper limbs, *VOR* vestibular-ocular reflex, *VUS* variant of uncertain significance, *w* wheelchair; *WES* whole exome sequencing, *WGS* whole genome sequencing

**Fig. 1:**
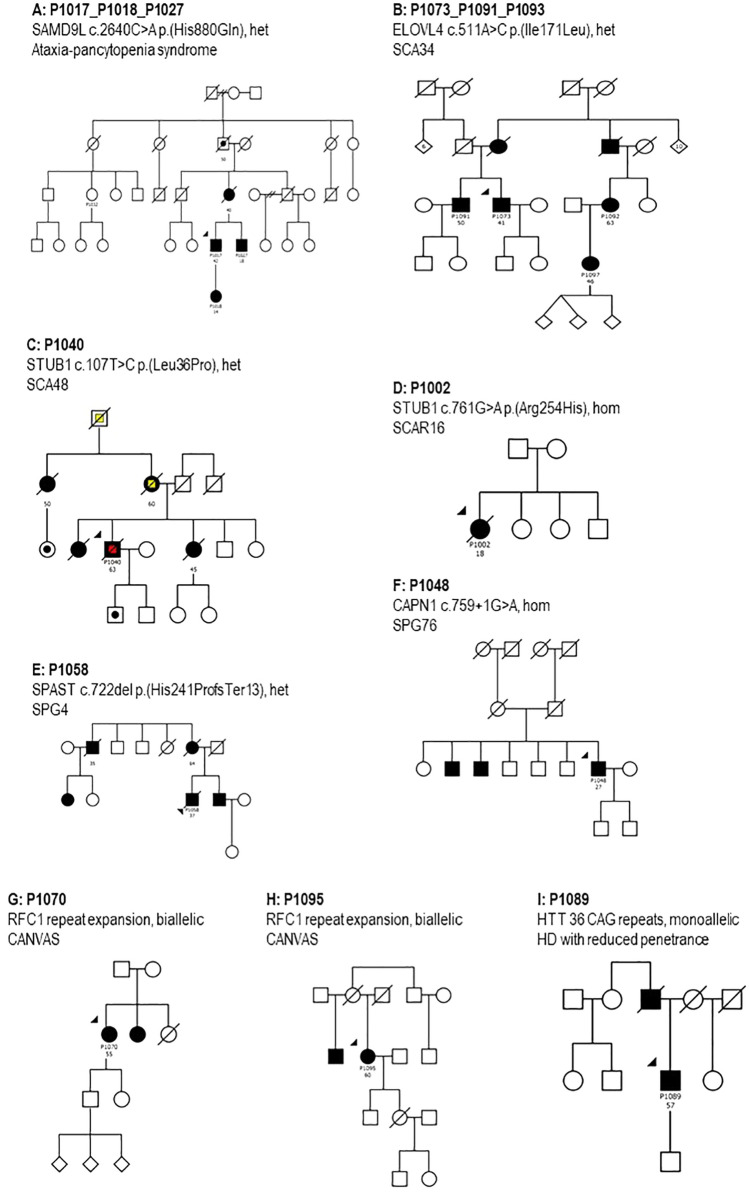

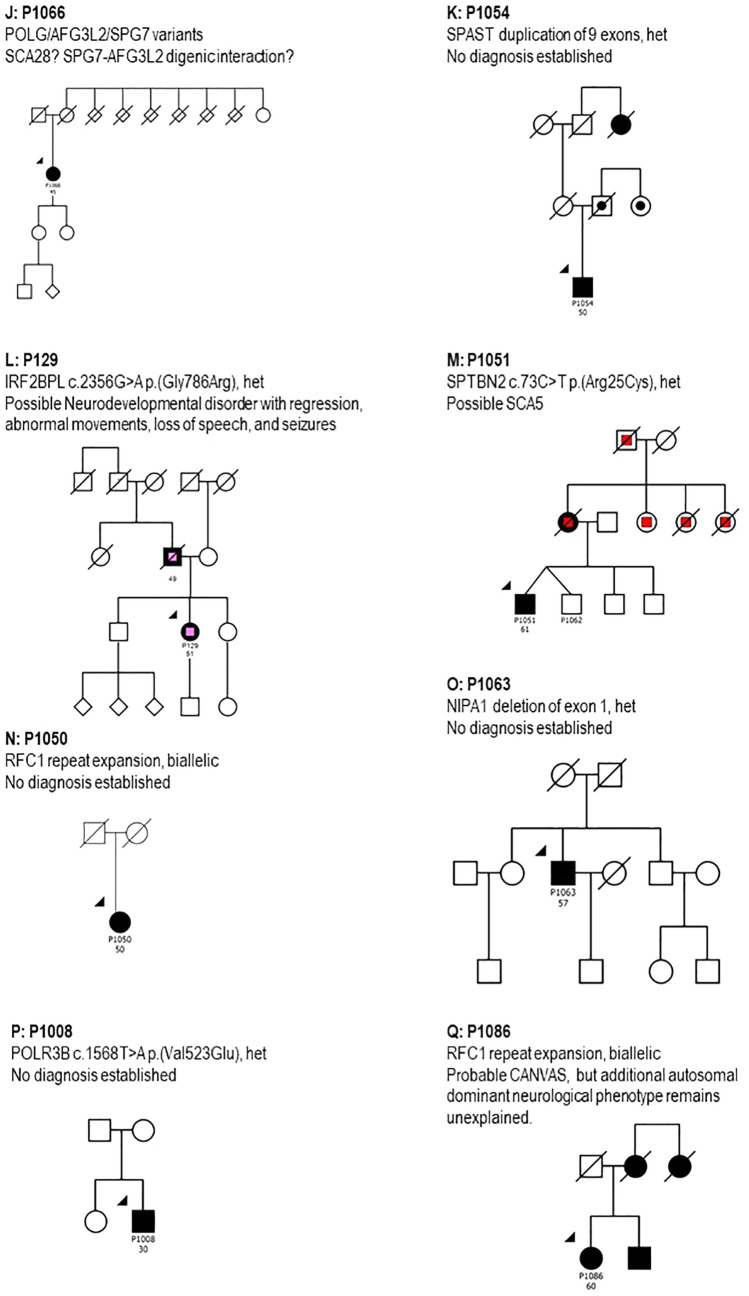
Pedigrees of patients and families examined genetically within this study. Round symbols indicate females, square symbols males; diagonal line indicates that the individual is deceased; patient identifiers and age at onset in years are provided below symbols; solid black symbols indicate ataxia, black dots indicate possible ataxia (acc. to family history); yellow color indicates possible dementia, red color indicates dementia. *CANVAS* cerebellar ataxia, neuropathy and vestibular areflexia; *HD* Huntington's disease, *het* heterozygosity, *hom* homozygosity, *SCA28* spinocerebellar ataxia 28, *SCA34* spinocerebellar ataxia 34, *SCA48* spinocerebellar ataxia 48, *SCA5* spinocerebellar ataxia 5, *SCAR16* autosomal recessive spinocerebellar ataxia 16, *SPG4* spastic paraplegia 4, *SPG7* spastic paraplegia 7, *SPG76* spastic paraplegia 76

**Fig. 2:**
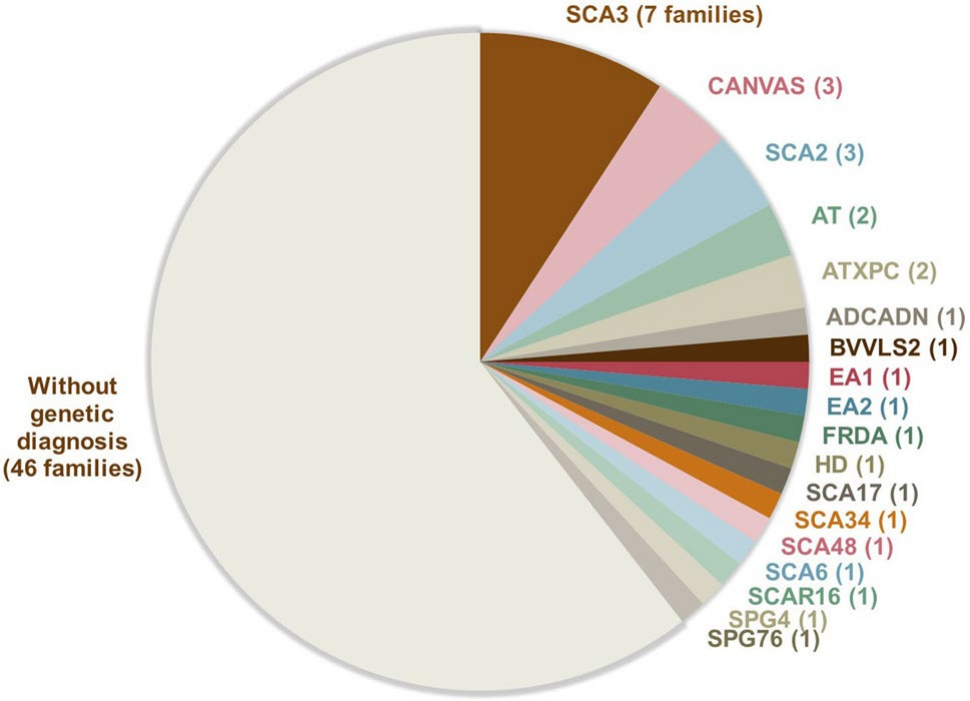
Subtypes of ataxia encountered in this study. Established molecular ataxia diagnoses in study participants as listed in Online Resources 1 and 2. Patients with SCA3 were recruited from a larger geographical area because they were included in multi-center studies on this disease. Nine additional patients with Friedreich ataxia from 7 families from our hospital’s uptake area had previously been included in another study and were not contacted again. *ADCADN* Autosomal dominant cerebellar ataxia, deafness, and narcolepsy, *AT* ataxia telangiectasia, *ATXPC* ataxia-pancytopenia syndrome, *BVVLS2* Brown–Vialetto–Van Laere syndrome-2, *CANVAS* cerebellar ataxia, neuropathy and vestibular areflexia, *EA* episodic ataxia, *FRDA* Friedreich’s ataxia, *HD* Huntington's disease, *SCA* spinocerebellar ataxia, *SCAR16* spinocerebellar ataxia autosomal recessive 16, *SPG* spastic paraplegia

Figure 1 shows the pedigrees of patients and families in Table 2 and Online Resource 2. Figure 2 summarizes the genetic diagnoses in this patient series. In the following paragraphs, we present clinical descriptions; additional and more detailed clinical descriptions are provided in Online Resource 3. The detailed phenotypes and pedigrees of index patients P1011 with ataxia pancytopenia syndrome and SAMD9L p.(Arg986Cys) variant and P1037 with Brown–Vialetto–Van Laere syndrome-2 have been published earlier [25, 26]. Patients were of Swedish origin unless stated otherwise.

*Index patient P1017* had heterozygous *SAMD9L* p.(His880Gln), and this variant had previously been reported in a large family with ataxia pancytopenia syndrome [27]. A diagnosis of ataxia pancytopenia syndrome was made. Clinical details are provided in Table 2 and Online Resource 3.

**Fig. 3:**
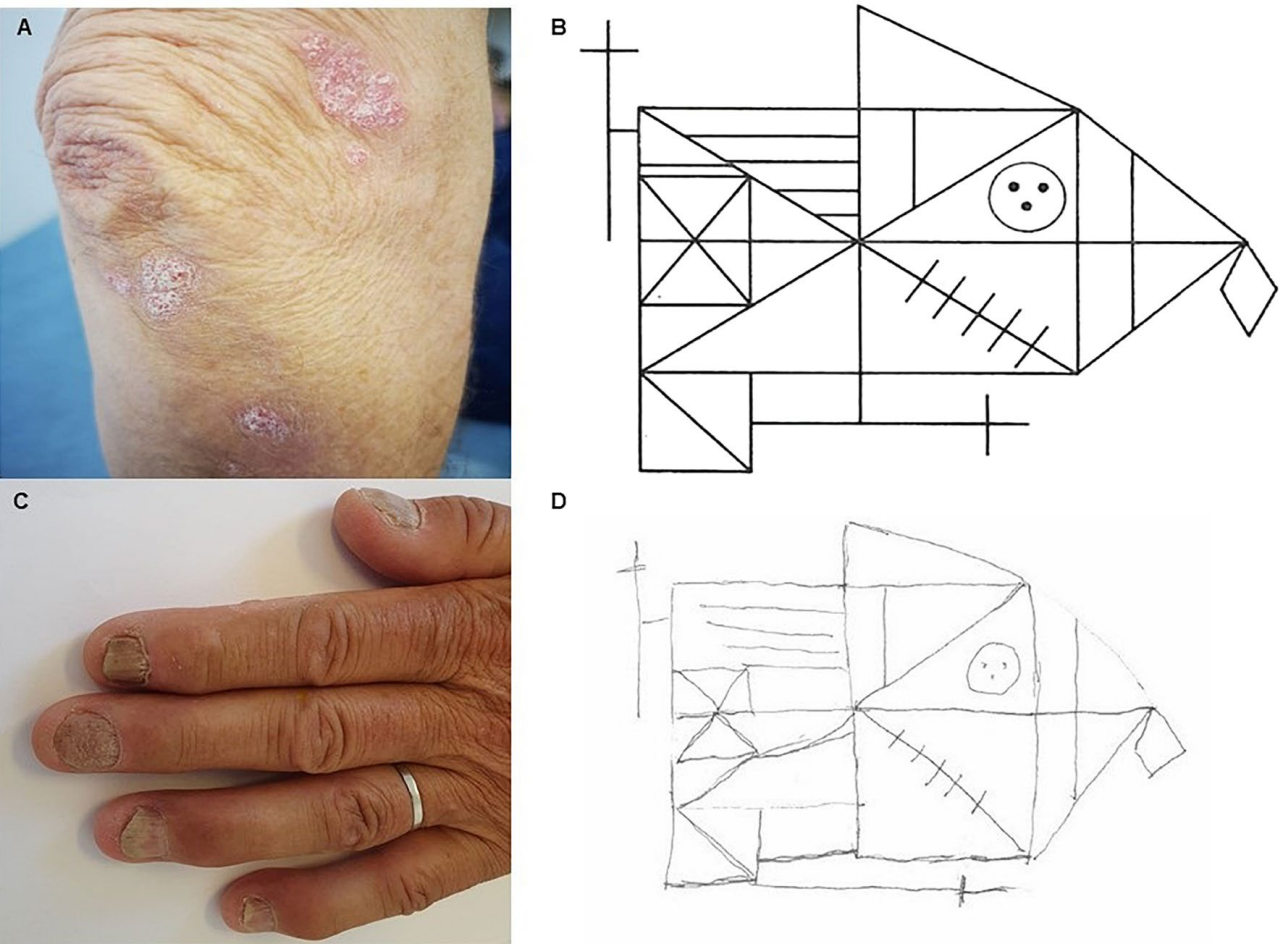
Images of clinical presentation in Spinocerebellar ataxia 34. **A**, **C** Hyperkeratotic skin manifestations present bilaterally on extensors and on the scalp and nail abnormalities in affected individuals which were diagnosed as psoriasis; **B**, **D** The Rey Complex Figure Test (RCFT) shows impaired visuo-spatial and executive function

*Index patient P1073* was referred to our clinic for investigation of ataxia with late onset. The patient reported that his brother P1091 had similar symptoms and that their mother and one of her brothers had balance impairment as well (Fig. 1B). This information suggested a form of autosomal dominant ataxia in the family. As a first step, the patient was analyzed clinically for the most common autosomal dominant spinocerebellar ataxias: SCA1, SCA2, SCA3, SCA6, SCA7, which was negative. Then, whole exome sequencing (WES) was performed for the analyses of an ataxia genes panel within the clinical workup, which also resulted in a negative outcome. Meanwhile, another relative of our index patient was investigated in parallel at the neurology clinic. She reported that her mother, who was the cousin P1092 of our index patient, had symptoms of balance impairment. The index patient P1073, his brother P1091, and the cousin P1092 expressed interest to be included in the ataxia research project. Blood samples from the brother and the cousin were sent to a different laboratory (Centogene, Rostock, Germany) which reported a variant of uncertain significance (VUS) in the *ELOVL4* gene c.511A > C, p.(Ile171Leu). No other findings were reported. Heterozygous variants in this gene are known causes of spinocerebellar ataxia type 34 (SCA34) [28, 29]. Clinical geneticists in Lund re-evaluated the existing genetic data from our index patient for the same variant that was found in his relatives, and it was confirmed positive and, in concordance with Centogene, initially classified as a VUS. After detailed post-NGS phenotyping of the three affected family members, we could conclude that all of them shared the typical clinical features for SCA34: decreased tendon reflexes, severe gait ataxia, gaze-evoked nystagmus, dysarthria, and hyperkeratotic skin manifestations (Fig. 3). Two individuals also had symptoms of cognitive decline with mildly to moderately impaired executive/visuospatial function [30]. Brain MRI showed moderate cerebellar atrophy for the index patient and severe cerebellar atrophy in his brother and cousin. Four affected family members carried the variant, and they were distant enough to each other in the pedigree to add evidence for the variant’s pathogenicity, using the modified ACMG criterion PP1_Strong for five informative meioses in the family, as suggested by Jarvik and Browning [31]. A reclassification as “likely pathogenic” according to the American College of Medical Genetics (ACMG) criteria was possible after the thorough family analyses for co-segregation of phenotype with genotype. We diagnosed SCA34.

*Index patient P1040* had a strong family history of dementia and balance impairment (Fig. 1C). Age at onset of neurological symptoms was in the mid-60s. Clinically the patient presented with gait impairment, severe ataxia in lower limbs and moderate ataxia in upper limbs, dysarthria, hypermetric saccades, motor restlessness, disinhibition, and perseverations. However, according to the patient’s next of kin, a slowly progressing personality change and mild cognitive impairment was noticed already in the early 50s. He was evaluated at our memory clinic and the clinical impression of his cognition and behavior was that of frontotemporal dementia. Brain MRI showed severe cerebellar and hippocampal atrophy. Genetic testing within our study identified a heterozygous variant in *STUB1* gene c.107T > C, p.(Leu36Pro) which was absent from population databases but had been reported to ClinVar (ClinVar ID 1297589) as a variant of uncertain significance. Heterozygous *STUB1* variants had been associated with spinocerebellar ataxia type 48 (SCA48). P1040 died during the study and was examined neuropathologically. Macroscopically, the patient had cerebellar atrophy, microscopically there was subtotal loss of Purkinje cells and atrophy of the molecular layer. The cerebrum showed tau-positive neurites, neuronal bodies, and astrocytes, with degenerative changes in the cortex and more pronounced degenerative changes in the thalamus, mesencephalon, and pons. There were p62-positive intraneuronal inclusions, as previously described for SCA48. *TBP* repeat length was normal (37 and 38 repeats, Online Resource 4). The patient’s clinical phenotype was very similar to patients with SCA48 described in the medical literature, and the unusual and specific histopathologic features in our patient were very well compatible with those described for SCA48 [32, 33].

*Index patient P1002*, of German extraction, had no family history of ataxia, but an early disease onset at 18 years of age which is suggestive of an autosomal recessive form of ataxia (Fig. 1D). Genetic testing within this study identified a homozygous variant in the *STUB1* gene, c.761G > A, p. (Arg254His) which previously had been reported as pathogenic. Biallelic pathogenic *STUB1* variants have been associated with autosomal recessive SCAR16 [34]. The patient died at the age of 42 years due to complications of her severe neurological disease.

*Index patient P1058* developed gait disturbance at 37 years of age and reported affected relatives in an autosomal dominant pattern of inheritance (Fig. 1E). At the time of the clinical examination, the patient had severe paraparesis, was wheelchair-bound, presented with lower limb ataxia (SARA score 13) and spasticity, inward rotation of the left foot, hyperreflexia, painful muscle cramps in thighs and calves. Brain CT was normal. Genetic analysis identified a heterozygous variant in the *SPAST* gene, c.722del, p.(His241Profs*13) previously reported as likely pathogenic. Pathogenic variants in the *SPAST* gene cause autosomal dominant spastic paraplegia 4 (SPG4) which we consider is in accordance with the clinical and genetic presentation of our patient.

*Index patient P1048* was of Turkish origin, with a family history suggestive of an autosomal recessive genetic condition; the patient reported that his two brothers were also affected but not the parents (Fig. 1F). The initial symptom was gait and balance impairment at the age of 27. On our examination, there was ataxia and spasticity in lower limbs, hyperreflexia, clonus, and bilateral positive Babinski sign. Brain MRI was normal. Genetic testing found a homozygous variant in the *CAPN1* gene, c.759 + 1G > A which has been previously described as pathogenic. Mutations in the *CAPN1* gene have been associated with spastic paraplegia 76 (SPG76) which was also confirmed for this patient [35].

*Index patients P1070 and P1095* are from two different families and developed symptoms at 55 and 60 years of age respectively. Both reported a family history suggestive of an autosomal recessive disease with siblings with similar symptoms (Fig. 1G/H). Analyses of WGS data for repeat expansion within our study revealed altered *RFC1* pentanucleotide composition (AAGGG instead of the normal AAAAG pentanucleotides) but were not able to reliably determine the number of pentanucleotides on each allele (Online Resource 4). Targeted testing using two orthogonal methods at an external laboratory confirmed biallelic extended repeat lengths in the *RFC1* gene, which has been previously described and associated with cerebellar ataxia with neuropathy and vestibular areflexia syndrome (CANVAS) [36]. CANVAS was diagnosed in both patients.

*Index patient P1089* (Fig. 1I) was referred to our neurology clinic because of a unilateral intention tremor in his right upper extremity. He reported that his father had developed a marked gait disturbance in his 80s and eventually required a wheelchair. Examination of P1089 by a movement disorder specialist at age 63 confirmed this and found moderate cerebellar tremor in the right hand and mildly atactic heel shin slide bilaterally, more pronounced on the right. Further, there was action and postural tremor of the essential tremor type (Online Resource 3) and mild dysarthria of the cerebellar type. This has led to the initial diagnosis of hereditary ataxia with late onset. During our clinical examination, he presented additional clinical signs such as irritability, cognitive impairment (MoCA 22/30 at age 64), tremor, and mild involuntary body movements. Brain MRI showed mild medial temporal atrophy. Genetic testing within our study showed 36 uninterrupted CAG trinucleotide repeats in the *HTT* gene (Online Resource 4). Alleles with 36–39 repeats usually have a low disease penetrance, however, normally the CAG repeats are interrupted by a CAA sequence in the penultimate 3′ triplet. Interestingly studies have shown that a loss of CAA interruption in *HTT* is associated with an earlier age at onset [37]. The initial symptom is described usually as a progressive cognitive decline, often associated with psychiatric problems [38]. The patient in our study did not have a loss of interruption. Based on the clinical presentation, the Huntington diagnosis was confirmed.

Genetic studies are still ongoing for some variants that were not yet confirmed as pathologic for specific cases, or it has remained impossible to fully elucidate pathogenicity. See Online Resource 2 and Fig. 1.

*Index patient P1008* reported no family history of ataxia or other neurologic disease (Fig. 1P). At age 30 years he started to experience impaired balance, impaired gait, and muscle weakness. Brain MRI showed signs of moderate cerebellar and lower brainstem atrophy. During the clinical examination within our study the following clinical signs were found: the patient was in a wheelchair, there were fasciculations of face muscles, moderate dysarthria, saccadic smooth pursuit, gaze-evoked nystagmus, general muscle atrophy, and distal weakness in upper and lower extremities, gynecomastia, myoclonus, hyperreflexia, and spasticity, bilateral positive Babinski signs, weight loss. Genetic analysis within our study identified a heterozygous variant in the *POLR3B* gene, c.1568 T > A, p.(Val523Glu). This variant was described as pathogenic in ClinVar. Pathogenic *POLR3B* variants are associated with autosomal dominant demyelinating Charcot-Marie-Tooth type 1 disease and autosomal recessive hypomyelinating leukodystrophy [39]. However, the patient’s particular variant has so far only been described in homozygous or compound heterozygous form in recessive disease. Our patient’s MRI images did not show any signs of hypomyelination and the patient did not report any family history of demyelinating polyneuropathy. The patient’s clinical phenotype was not well compatible with the *POLR3B*-associated diseases. We conclude that the disease’s cause remains unknown.

*Index patient P1086* had a family history of autosomal dominant motor polyneuropathy and an age at onset of 60 years. (Fig. 1Q). The patient had gait and limb ataxia, sensorimotor polyneuropathy, impaired vibration sense, hypometric saccades, hypoactive vestibulo-ocular reflex, hyperreflexia, and tremor of the head and left hand. Brain MRI showed mild cerebellar atrophy and white matter lesions. Similar to P1070 and P1095 described above, extended repeat lengths were found in the *RFC1* gene. Nevertheless, we remain uncertain about this finding since CANVAS is an autosomal recessive disease and this patient has a family history of autosomal dominant motor polyneuropathy. The evaluation of genes known to cause hereditary polyneuropathies by our bioinformaticians was negative. The vestibular signs and the polyneuropathy are compatible with the CANVAS diagnosis, but the hyperreflexia is not easily explained. Our present hypothesis is that this patient may have CANVAS but also an additional dominantly inherited disorder.

## Discussion

This study describes a series of 87 patients with progressive ataxia actively recruited within the uptake area of Skåne University Hospital over a period of 8 years and illustrates the process of examining patients with progressive ataxia with or without a confirmed genetic diagnosis. Our results show the high variability between the phenotypes of different forms of ataxia and the complexity of the interpretation of genetic findings.

Our study sheds some light on the presence of genetic forms of ataxia in the Swedish population. The most common form of autosomal dominant ataxia in our patient series was SCA3, which may be explained by the fact that we actively searched for and recruited these patients for a multi-centre study on biomarkers in SCA3 [13, 14]. Another more common form of autosomal dominant ataxias in our series was SCA2. These findings are similar to what was previously reported for European populations [6, 40, 41]. We found CANVAS to be the most common autosomal recessive form of ataxia among our patients, in line with other studies confirming that biallelic AAGGG expansion in *RFC1* is a frequent cause of late-onset ataxia in Europeans [36]. Friedreich ataxia may be more prevalent, but most patients had been included in an earlier research study. Several rarer genetic forms of ataxia were also encountered, as expected with the increasingly large number of internationally known diseases with ataxia. Additionally, we found diagnoses that are not classically counted as genetic ataxias; two patients had hereditary spastic paraplegia, one Huntington’s disease and one Brown–Vialetto–Van Laere syndrome-2. Although this might be explained by the fact that clinicians registered the “wrong” ICD-10 code for these patients, all patients did have ataxia on examination, and hereditary ataxia and hereditary spastic paraplegias share not only overlapping phenotypes and underlying genes but also common disease mechanisms and cellular pathways [42]. Ataxia has been reported as an initial finding in 8.3% of patients with Huntington’s disease, and in over 70% during later disease stages [43]. The presence of these disorders among patients with a clinical diagnosis of ataxia shows that grouping neurological disorders with combined symptomatology can be difficult, or impossible, as the clinically most prominent symptom or finding may change over time or vary between individuals with the same genetic disease cause.

NGS technology has seen a rapid development and refinement, also during the time of our study. Sophisticated algorithms have been developed for the bioinformatic filtering of the ample sequencing data that result from NGS methods. Increasingly accurate indirect bioinformatic methods also detect copy number variants or short tandem repeats in new-generation sequencing datasets; these types of genetic variants are not directly assessed by sequencing. In this study, we used WES and WGS and analyzed the data for single nucleotide variants and small insertions or deletions, for copy number variants and short tandem repeats. We chose WES initially for reasons of cost and availability at the time, but transitioned to WGS, in part motivated by the discovery that intronic *RFC1* variants were a relatively common cause of recessive ataxia with a particular combination of signs and symptoms [36]. The bioinformatic analyses detected an expansion of the *HTT* gene (36 trinucleotide repeats, total length 108 nucleotides) and unambiguously determined the repeat expansion’s length. Our methodology failed to reliably determine the length of the *RFC1* pentanucleotide repeats based on our short-read WGS data. However, we detected the known abnormality in the base sequence of the pentanucleotides associated with *RFC1* expansions and in all cases where we continued to analyze the patients by additional methods, expansions were identified. Long-read sequencing is likely to become more widely available in the near future and is expected to accurately determine the length of longer repeat expansions with thousands of repeat units [23]. Clearly, the use of short read WES rather than WGS and/or long-read sequencing is a limitation of our study, but our study used the methods that are presently available for clinical diagnostics in many healthcare settings. It is difficult to estimate how many additional patients may have received a diagnosis had they been examined by the more advanced NGS methods rather than short-read WES.

Eleven families received a genetic diagnosis during our study (Table 2). We considered two variants to be the disease cause that according to ACMG guidelines were classified only as variants of uncertain significance when they were first detected: Heterozygous *ELOVL4* NM_022726.4 c.511A > C p.(Ile171Leu) in Family P1073_P1091_P1093 (Fig. 1B) and heterozygous *STUB1* NM_005861.2 c.107 T > C p.(Leu36Pro) in familial proband P1040 (Fig. 1C). The *ELOVL4* variant was present in four affected family members; two siblings, their first-degree cousin and that cousin’s daughter, which increased the likelihood for its pathogenicity according to predefined criteria [31]. Also, there was a characteristic combination of ataxia and skin and nail changes (“psoriasis” and hyperkeratotic nails) as well as a mild visuospatial and executive function deficit, of the same type as previously describe in this disease [28–30]. The patient with the heterozygous *STUB1* variant showed an unusual clinical combination of ataxia with frontotemporal cognitive dysfunction, and the histo-neuro-pathological examination showed unusual inclusions that are very well compatible with the diagnosis SCA48 [32]. The PP4 criterium in the ACMG guidelines can be used when “the patient has a well-defined syndrome with little overlap with other clinical presentations”, such as in these two cases, but PP4 only counts as “supporting” the pathogenicity. For very rare and almost pathognomonic combinations of clinical features, as in these very rare neurological disorders, the weight of this criterium may be increased. However, there have also been concerns that the presence of both genetic and phenotypic diversity and the general “narrative potential” of a human genome [44] may lead to overinterpretation and wrong conclusions regarding variants of uncertain significance, especially in a post-NGS phenotyping scenario.

Recent work reported di-genic inheritance of intermediate length *TBP* expansions (40–49 repeats) and heterozygous *STUB1* variants [45]. In almost all families, only individuals who carried variants in both these genes developed the clinical phenotype, that was named SCA17-DI (for di-genic) and who were observed to share certain clinical features [46]. This observation was partially replicated in a larger case series but only about half the patients with SCA17 and intermediate length *TBP* expansions had *STUB1* variants, and more than a third of patients with SCA48 had normal *TBP* repeat size [47], as our patient with SCA48 (P1040). We have unfortunately been unable to examine *STUB1* in our patient with SCA17 (P1047) in whom an intermediate length *TBP* allele was found several years ago during clinical testing.

For 8 patients, we had seemingly relevant genetic findings but were not able to set a clear diagnosis for variable reasons (Online Resource 2). In some of these families, examination of the proband’s relatives may lead to a diagnosis. We also encountered the situation that a patient (P1086, Online Resource 2) had a confirmed, clearly pathogenic variant (*RFC1* repeat expansion), but we remained doubtful if this could explain the full clinical picture of this patient and the family.

Most of the patients included in our study are being followed at our clinic and we thus routinely re-evaluate new ways to provide a diagnosis. This includes testing for newly discovered genetic causes for ataxia that so far evade detection in WES or WGS analyses, re-running bioinformatic analyses with updated gene lists, and perhaps in the near future also new methods such as long-read sequencing, considering di-genic inheritance. We try to expand family pedigrees and test additional informative family members, when contact with these can be established. Likely, the majority of patients have a genetic cause for their ataxia, but non-genetic causes that have not been assessed or that may not yet be known cannot entirely be excluded.

Additional limitations of our study include the relatively small number of participants, which however is a common challenge for research on rare diseases. A proportion of the 158 contacted were not included in the present analyses, because they declined participation or because of scheduling difficulties. Our results are based on a selected patient cohort, and thus may not represent the true prevalence of genetic ataxia subtypes in Sweden. The study design reflects that of a real-life diagnostic clinic and neurologists were not blinded for the genetic results but were also informed about potentially relevant variants of uncertain significance. This could lead to false-positive results, but we consider the risk for this low, at least in the setting of a research study at a highly specialized center, and rather see the advantages of multi-disciplinary discussions to obtain a diagnosis, as is common practice in many other (non-genetic) diagnostic situations.

## Conclusion and outlook

Analysis of our case series confirms that progressive ataxias have many different causes even when patients come from a relatively small geographic area. NGS sequencing is a powerful tool in clinical diagnostics; the absence of family history should not exclude genetic testing in patients with progressive ataxia. Re-examination of NGS datasets, as new diseases are being described, leads to more diagnoses. Closer clinical examination of patients and families with high-suspicion variants of uncertain significance may lead to additional correct diagnoses [48, 49], but clinical findings need to be re-assessed critically to confirm or reject a diagnosis [44]. The task lies ahead to define if and how this workflow can broadly be implemented in clinical diagnostics in healthcare settings, or if our approach will need to remain confined to research studies such as this one.

The ACMG diagnostic criteria [21] are helpful and, however, have their own limitations when applied for very rare diseases. Further studies may evolve these criteria that have remained unchanged since 2015 and determine new ways to combine neurological and genetic experience and knowledge from both clinical and research analyses, so that more patients will receive a genetical diagnosis within their workup in a healthcare setting.

### Supplementary Information

Below is the link to the electronic supplementary material.

Supplementary file1 (DOCX 1122 KB)

## Data Availability

According to Swedish law and national, regional and institutional regulations, individual-level genetic data can only under very special circumstances be made available to collaborators. Reasonable requests can be directed to the corresponding author (SG).
