# Supplementary material for: Clinical and genetic analyses of a Swedish patient series diagnosed with ataxia
Source: J Neurol. 2023 Oct 3;271(1):526–42. doi: 10.1007/s00415-023-11990-x (PMC10770240; doi:10.1007/s00415-023-11990-x)
Supplement: Supplementary file 1 — Supplementary file1 (DOCX 1122 KB) [file 415_2023_11990_MOESM1_ESM.docx]

**Online Resources**

***Clinical and genetic analyses of a Swedish patient series diagnosed with ataxia***

*Sorina Gorcenco, Efthymia Kafantari, Joel Wallenius, Christin Karremo, Erik Alinder, Sigurd Dobloug, Maria Landqvist Waldö, Elisabet Englund, Hans Ehrencrona, Klas Wictorin, Kristina Karrman, Andreas Puschmann*

*Journal of Neurology*

**Content:**

[Online Resource 1 (table): Study patients who had received a genetic diagnosis through clinical analyses 2](#_Toc143547886)

[Online Resource 2 (table): Study patients with rejected or uncertain genetic findings or where family co-segregation analysis is pending 9](#_Toc143547887)

[Online Resource 3 (text): Additional/more detailed clinical vignettes 12](#_Toc143547888)

[Online Resource 4 (figure): Analyses of *RFC1* pentanucleotide and *HTT* and *TBP* trinucleotide expansions 14](#_Toc143547889)

[References to Online Resources 18](#_Toc143547890)

# Online Resource 1 (table): Study patients who had received a genetic diagnosis through clinical analyses

| **Proband only / Family** | **Patient ID** | **Cerebellar signs / Pyramidal signs / Other clinical details** | **Gene(s), Transcript(s), Variant(s), Genotype** | **Database information (Manual variant classification by Clinical geneticist)** | **Genetic diagnosis (Comment)** |
| --- | --- | --- | --- | --- | --- |
| Proband only | P1015 | AO 1yrs. SD 39yrs. Gait (w), UL, LL, ataxia. Dysarthria. Horizontal nystagmus, saccadic smooth pursuit, hypometric and slow saccades, omnidirectional ophthalmoplegia. SARA 28. SARA/SD=0.72/y. MRI/CT: ND.  No pyramidal signs.  Impaired visual acuity; areflexia, muscle weakness in LL, impaired vibration sense. | *ATM* NM_000051  c.9029T>G p.(Leu3010*) /  exon 43 deletion  comp het | ClinVar: NR / Pathogenic (ID 663959)  gnomAD NFE genomes & exomes; Swefreq: Absent / NR  CADD-phred: 40 / NR  Varsome ACMG: VUS  Franklin by Genoox: Likely pathogenic | Ataxia telangiectasia. |
| Proband only | P1046 | AO 1yrs. SD 46yrs. Gait (w), UL+LL ataxia. Dysarthria. Saccadic & low gain pursuit, downbeat & horizontal nystagmus, hypometric saccades, hypoactive VOR. SARA 37. SARA/SD=0.80/y. MRI/CT: ND.  Clonus, EPR.  Anxiety, depression, bradykinesia, LL impaired sensibility & weakness, finger contractures. | *ATM* NM_000051  c.487C>T p.(Gln163*) /  c.3284G>C p.(Arg1095Thr)  comp het | ClinVar: Pathogenic / VUS  gnomAD NFE genomes & exomes; Swefreq: Absent / Absent  CADD-phred: 40/32  Varsome ACMG: Pathogenic/VUS  Franklin by Genoox: Pathogenic/VUS | Ataxia telangiectasia. |
| Proband only | P1003 | AO 5yrs. SD 54yrs. Gait & LL ataxia. SARA 3. SARA/SD=0.06/y. MRI/CT: ND.  No pyramidal signs.  Cognitive impairment, myotonia in calves, hands, eyelids, tongue, fasciculations, muscle cramps, tinnitus, hypoacusis. | *KCNA1* NM_000217.2  c.520G>T p.(Val174Phe)  het | ClinVar: Pathogenic (ID 13482)  gnomAD NFE genomes & exomes; Swefreq: Absent  CADD-phred: 26.7  Varsome ACMG: Likely pathogenic  Franklin by Genoox: Likely pathogenic | Episodic ataxia 1.  (Functional data on this variant is available; it results in a rightward shift in the voltage-dependent activation of this potassium channel [1]) |

| Family_  P1012_  P1019_  P1024 | P1012 (proband’s father) | AO ND. SD ND. Gait ataxia. Dysarthria: ND. SARA: ND. MRI/CT: severe CA, brainstem & medial temporal lobe atrophy.  Pyramidal signs: ND.  Other signs: ND. | *CACNA1A* NM_001127221.1  c.1482_1483del p.(Ser495fs)  het  P1012, P1019 and P1024 carry this variant.  P1024’s sister and brother (both have no ataxia) do not carry the *CACNA1A* variant. | ClinVar: NR  gnomAD NFE genomes & exomes; Swefreq: Absent  CADD-phred: NR  Varsome ACMG: Pathogenic  Franklin by Genoox: Pathogenic | Episodic ataxia 2. |
| --- | --- | --- | --- | --- | --- |
|  | P1019 (proband’s daughter) | AO 15yrs, SD 21yrs (for vertigo that episodically worsens). No ataxia on examination. SARA 0. MRI/CT: ND.  Hyperreflexia. Vertigo. ADHD, depression, hypersomnia. LL muscle weakness, UL paresthesias, orthostatic hypotension. |  |  |  |
|  | P1024 (proband) | AO 10yrs. SD 51yrs. Gait ataxia. SARA 3. SARA/SD=0.06/y. MRI/CT: ND. GEN. Low gain smooth pursuit, saccadic pursuit.  No pyramidal signs.  Areflexia. Mild cognitive impairment, mild dysphagia, impaired visual acuity, mild urinary dysfunction. |  |  |  |
| Proband only | P1087 | AO 34yrs. SD 10yrs. Gait & UL ataxia. SARA 5. SARA/SD=0.50/y. Saccadic pursuit, horizontal nystagmus. MRI/CT: moderate CA, moderate temporo-occipital & corpus callosum atrophy, severe frontoparietal atrophy, empty sella.  EPR, UL hyperreflexia.  LL areflexia, hypoacusis, bilateral hand tremor, depression, kidney disease, high blood pressure. | *DNMT1* NM_001130823.1  c.1816G>T p.(Val606Phe)  het | ClinVar: NR  gnomAD NFE genomes & exomes; Swefreq: Absent  CADD-phred: 28  Varsome ACMG: Likely pathogenic  Franklin by Genoox: VUS  Manual ACMG: (Likely pathogenic: PM1 + PM2 + PP1 + PP5. AD condition.) | Autosomal dominant cerebellar ataxia, deafness, and narcolepsy.  (Patient belongs to a Swedish kindred where this mutation had previously been described [2] and [3].) |

| Family  P1093_  P1094 | P1093 sibling 1 | AO 14yrs. SD 10yrs. Gait (w), UL & LL ataxia. Dysarthria. SARA 27. SARA/SD=2.70/y. Horizontal nystagmus, high gain VOR. Hypometric saccades, saccadic and high gain smooth pursuit. MRI/CT: mild CA.  No pyramidal signs.  Areflexia, sensory neuropathy, UL & LL weakness, scoliosis, depression. | *FXN* NM_000144.5  trinucleotide repeat expansion  monoallelic | ClinVar: Pathogenic  gnomAD NFE genomes & exomes; Swefreq: NA  CADD-phred: NA | Friedreich ataxia. |
| --- | --- | --- | --- | --- | --- |
|  | P1094 sibling 2 | AO 15yrs. SD 12yrs. Gait (w), UL & LL ataxia. Dysarthria. SARA 25.5. SARA/SD=2.13/y. Low gain and saccadic smooth pursuit, depression, GEN, hypometric saccades. MRI/CT: normal.  No pyramidal signs.  Areflexia, sensory neuropathy, weakness LL, history of head trauma, insomnia. |  |  |  |
| Proband only | P1031 | AO 53yrs. SD 8yrs. Gait, UL & LL ataxia. SARA 10. SARA/SD=1.25/y. Hypometric & slow saccades, saccadic & low gain pursuit. MRI/CT: moderate CA.  No pyramidal signs.  Urinary dysfunction. | *ATXN2* NM_001372574.1  trinucleotide repeat expansion  monoallelic | ClinVar: Pathogenic  gnomAD NFE genomes & exomes; Swefreq: NA  CADD-phred: NA | Spinocerebellar ataxia 2. |
| Proband only | P1045 | AO 36yrs. SD 7yrs. Gait, UL & LL ataxia. Dysarthria. SARA 6. SARA/SD=0.86/y. MRI/CT: severe cerebellar & brainstem atrophy.  No pyramidal signs.  UL & LL muscle cramps, UL tremor, LL weakness & decreased sensibility; fatigue, dizziness, depression, insomnia. | *ATXN2* NM_001372574.1  trinucleotide repeat expansion  monoallelic | ClinVar: Pathogenic  gnomAD NFE genomes & exomes; Swefreq: NA  CADD-phred: NA | Spinocerebellar ataxia 2. |

| Family  P1036_  P1060 | P1036 | AO 38yrs. SD 12yrs. Gait, UL & LL ataxia. Dysarthria. SARA 11.5. SARA/SD=0.96/y. Slow saccades. MRI/CT: moderate CA.  No pyramidal signs.  Hyporeflexia, UL & LL muscle cramps. | *ATXN2* NM_001372574.1  trinucleotide repeat expansion  monoallelic | ClinVar: Pathogenic  gnomAD NFE genomes & exomes; Swefreq: NA  CADD-phred: NA | Spinocerebellar ataxia 2. |
| --- | --- | --- | --- | --- | --- |
|  | P1060 | AO 60yrs. SD 15yrs. Gait, UL & LL ataxia. Dysarthria. SARA 14. SARA/SD=0.93/y. Low gain & saccadic pursuit, slow & hypometric saccades, bilateral upward gaze palsy. MRI/CT: moderate CA.  No pyramidal signs.  Hyporeflexia, tremor, UL muscle weakness, muscle atrophy, LL muscle cramps, decreased vibration sense, face/neck/UL&LL dystonia. Cognitive impairment, depression. |  |  |  |
| Proband only | P1029 | AO 28yrs. SD 30yrs. Gait (w), UL & LL ataxia. Dysarthria. SARA 33.5. SARA/SD=1.12/y. Saccadic intrusions, horizontal nystagmus, hypometric saccades. MRI/CT: ND.  No pyramidal signs.  Scoliosis, unilateral ptosis. | *ATXN3* NM_004993.6  trinucleotide repeat expansion  monoallelic | ClinVar: Pathogenic  gnomAD NFE genomes & exomes; Swefreq: NA  CADD-phred: NA | Spinocerebellar ataxia 3. |
| Proband only | P1033 | AO 45yrs. SD 7yrs. Gait, UL & LL ataxia. Dysarthria. SARA 13. SARA/SD=1.86/y. Hypermetric saccades, saccadic pursuit, GEN, square wave jerks on fixation, hyperactive VOR. MRI/CT: mild CA.  No pyramidal signs.  Sensorimotor neuropathy, impaired vibration sense, urinary dysfunction, UL+LL dystonia. Hallucinations & delusions, depression. History of head trauma. | *ATXN3* NM_004993.6  trinucleotide repeat expansion  monoallelic | ClinVar: Pathogenic  gnomAD NFE genomes & exomes; Swefreq: NA  CADD-phred: NA | Spinocerebellar ataxia 3. |
| Proband only | P1041 | AO 32yrs. SD 16yrs. Gait UL+LL ataxia. Dysarthria. SARA 14. SARA/SD=0.86/y. Horizontal nystagmus, slow and hypometric saccades, hypoactive VOR. MRI/CT: normal.  No pyramidal signs.  Areflexia, decreased sensation for temperature & vibration, LL muscle cramps, urinary dysfunction, diplopia, bradykinesia, cognitive impairment. | *ATXN3* NM_004993.6  trinucleotide repeat expansion  monoallelic | ClinVar: Pathogenic  gnomAD NFE genomes & exomes; Swefreq: NA  CADD-phred: NA | Spinocerebellar ataxia 3. |
| Family  P1016_  P1042 | P1016 (proband) | AO 44yrs. SD 7yrs. Gait UL ataxia. Dysarthria. SARA 11. SARA/SD=1.57/y. Horizontal nystagmus, slow & hypometric saccades, saccadic smooth pursuit, ophthalmoparesis on vertical gaze. MRI/CT: ND.  No pyramidal signs.  Areflexia, impaired proprioception, impaired vibration sense, decreased sense for pain in LL , fasciculations. UL & LL dystonia, hypokinesia, LL rigidity, tremor, vertigo, RBD. Depression, double vision, dysphagia. | *ATXN3* NM_004993.6  trinucleotide repeat expansion  monoallelic | ClinVar: Pathogenic  gnomAD NFE genomes & exomes; Swefreq: NA  CADD-phred: NA | Spinocerebellar ataxia 3. |
|  | P1042 (proband’s child) | No ataxia. SARA 0. MRI/CT: ND.  No pyramidal signs.  Orthostatic hypotension, depression. | *ATXN3* NM_004993.6  trinucleotide repeat expansion  monoallelic | ClinVar: Pathogenic  gnomAD NFE genomes & exomes; Swefreq: NA  CADD-phred: NA | Asymptomatic carrier. |
| Family  P1023_  P1044 | P1023 | AO 38yrs. SD 4yrs. Gait, UL & LL ataxia. Dysarthria. SARA 8. SARA/SD=2.00/y. MRI/CT: ND.  Hyperreflexia.  Blepharospasm, writers’ cramp, scoliosis. Orthostatic hypotension, LL paresthesias, urinary dysfunction, fasciculations. Double vision, fatigue, dysphagia, RBD, history of head trauma. | *ATXN3* NM_004993.6  trinucleotide repeat expansion  monoallelic | ClinVar: Pathogenic  gnomAD NFE genomes & exomes; Swefreq: NA  CADD-phred: NA | Spinocerebellar ataxia 3. |
|  | P1044 | AO 34yrs. SD 7yrs. Gait & LL ataxia. Dysarthria. SARA 5. SARA/SD=0.71/y. Hypometric & slow saccades. MRI/CT: ND.  Hyperreflexia.  Diplopia, esotropia, urinary retention, restless legs. Dysphagia, weight loss, fatigue, insomnia. | *ATXN3* NM_004993.6  trinucleotide repeat expansion  monoallelic | ClinVar: Pathogenic  gnomAD NFE genomes & exomes; Swefreq: NA  CADD-phred: NA | Spinocerebellar ataxia 3. |

| Family  P1026_  P1028_  P1030 | P1026 (proband) | AO 30yrs. SD 29yrs. Gait (w),UL & LL ataxia. Dysarthria. SARA 31. SARA/SD=1.07/y. Low-gain and saccadic smooth pursuit., hypometric saccades. MRI/CT: ND.  No pyramidal signs.  Areflexia, UL & LL muscle weakness, LL muscle cramps, urinary dysfunction. Insomnia, RBD, hallucinations & delusions. | *ATXN3* NM_004993.6  trinucleotide repeat expansion  monoallelic | ClinVar: Pathogenic  gnomAD NFE genomes & exomes; Swefreq: NA  CADD-phred: NA | Spinocerebellar ataxia 3. |
| --- | --- | --- | --- | --- | --- |
|  | P1028 (proband’s brother) | AO 39yrs. SD 14yrs. SARA/SD=0.36/y. Gait, UL & LL ataxia. Dysarthria. SARA 19. Hypometric saccades. MRI/CT: ND.  No pyramidal signs.  Areflexia, muscle cramps LL, impaired vibration sense. | *ATXN3* NM_004993.6  trinucleotide repeat expansion  monoallelic | ClinVar: Pathogenic  gnomAD NFE genomes & exomes; Swefreq: NA  CADD-phred: NA | Spinocerebellar ataxia 3. |
|  | P1030  (proband’s child) | AO 25yrs. SD 16yrs. SARA/SD=0.64/y. Gait, UL & LL ataxia. Dysarthria. SARA 8. Hypometric saccades. MRI/CT: ND.  LL spasticity, hyperreflexia.  Bradykinesia. | *ATXN3* NM_004993.6  trinucleotide repeat expansion  monoallelic | ClinVar: Pathogenic  gnomAD NFE genomes & exomes; Swefreq: NA  CADD-phred: NA | Spinocerebellar ataxia 3. |
| Family  P1043_  P1049 | P1043 (proband) | AO 30yrs. SD 14yrs. Gait, UL & LL ataxia. Dysarthria. SARA 9. SARA/SD=0.64/y. Slow saccades, low gain & saccadic pursuit, hyperactive VOR. MRI/CT: ND.  Hyperreflexia.  Orthostatic hypotension, bradykinesia, rigidity, resting tremor. Insomnia. | *ATXN3* NM_004993.6  trinucleotide repeat expansion  monoallelic | ClinVar: Pathogenic  gnomAD NFE genomes & exomes; Swefreq: NA  CADD-phred: NA | Spinocerebellar ataxia 3. |
|  | P1049  (proband’s sister) | No ataxia. SARA 0. MRI/CT: ND.  Hyperreflexia.  Insomnia, fasciculations. | *ATXN3* NM_004993.6  trinucleotide repeat expansion  monoallelic | ClinVar: Pathogenic  gnomAD NFE genomes & exomes; Swefreq: NA  CADD-phred: NA | Asymptomatic carrier. |
| Proband only | P1014 | AO 35yrs. SD 4yrs. Gait & LL ataxia. SARA 2. SARA/SD=0.50/y. MRI/CT: ND.  No pyramidal signs.  Areflexia, sensorimotor polyneuropathy, urinary retention, impaired vibration sense, impaired visual acuity. | *CACNA1A* NM_001127221.1  trinucleotide repeat expansion  monoallelic | ClinVar: Pathogenic  gnomAD NFE genomes & exomes; Swefreq: NA  CADD-phred: NA | Spinocerebellar ataxia 3.  (Genetically analysed at a different hospital; original report not available). |

| Proband only | P1047 | AO 17yrs. SD 5yrs. Gait, UL & LL ataxia. SARA 6.5. SARA/SD=1.30/y. Low gain & saccadic smooth pursuit. MRI/CT: normal.  No pyramidal signs.  UL+LL dystonia, UL tremor. Depression, insomnia. | *TBP* NM_003194.5  trinucleotide repeat expansion  43 +/- 1 repeats  monoallelic | ClinVar: Pathogenic with reduced penetrance  gnomAD NFE genomes & exomes; Swefreq: NA  CADD-phred: NA | Spinocerebellar ataxia 17. |
| --- | --- | --- | --- | --- | --- |
| Family  P1079_  P1084 | P1079 (proband) | AO 65yrs. SD 7yrs. Gait, UL & LL ataxia. Dysarthria. SARA 20. SARA/SD=2.86/y. GEN, low gain & saccadic smooth pursuit, hypometric saccades, hypoactive VOR. MRI/CT: mild CA, widespread white matter lesions.  Hyperreflexia.  Sensorimotor neuropathy, impaired sensibility for temperature & vibration and impaired proprioception, urinary dysfunction. | *RFC1* NM_002913.5  pentanucleotide repeat expansion  biallelic | ClinVar: Pathogenic  gnomAD NFE genomes & exomes; Swefreq: NA  CADD-phred: NA | Cerebellar ataxia, neuropathy, and vestibular areflexia syndrome. |
|  | P1084 (proband’s cousin) | AO 58yrs. SD 10yrs. Gait, UL & LL ataxia. Dysarthria. SARA 10. SARA/SD=1.00/y. MRI/CT: normal. No pyramidal signs. sensory neuropathy, orthostatic hypotension, muscle cramps LL, unilateral ptosis, impaired sensibility for temperature UL+LL, hypersomnia, | *RFC1* NM_002913.5  pentanucleotide repeat expansion  biallelic | ClinVar: Pathogenic  gnomAD NFE genomes & exomes; Swefreq: NA  CADD-phred: NA | Cerebellar ataxia, neuropathy, and vestibular areflexia syndrome. |

**Clinical phenotype and genetic findings in patients with a known genetic diagnosis**

AD - autosomal dominant; ADHD - attention-deficit/hyperactivity disorder; AO - age at onset; AR - autosomal recessive; CA - cerebellar atrophy; CADD-phred - combined annotation dependent depletion; a tool for scoring the deleteriousness of single nucleotide variants as well as insertion/deletions variants in the human genome; comp het - compound heterozygosity ; CT - computed tomography; EPR - extensor plantar response; GEN - gaze-evoked nystagmus; het - heterozygosity; LL - lower limbs; MRI - magnetic resonance imaging; NA – not available; ND - not determined; NFE - Non-Finnish-European; NR - not reported; RBD - REM sleep behavior disorder; SARA - scale for assessment and rating of ataxia; SD - symptom duration (time from symptom onset to examination within the study); UL - upper limbs; VOR - vestibular-ocular reflex; VUS - variant of uncertain significance; w - wheelchair

# Online Resource 2 (table): Study patients with rejected or uncertain genetic findings or where family co-segregation analysis is pending

| **Patient ID** | **Cerebellar signs / Pyramidal signs / Other clinical details** | **Gene(s), Transcript(s), Variant(s), Genotype, Test method(s)** | **Database information (Manual variant classification by Clinical geneticist)** | **Genetic diagnosis (Comment)** |
| --- | --- | --- | --- | --- |
| P1066  (Fig. 1J) | AO 45yrs. SD 13yrs. Gait UL+LL ataxia. SARA 8. SARA/SD=0.62/y. Horizontal nystagmus. MRI/CT: normal.  Hyperreflexia.  Hearing loss, bilateral severe ptosis, muscle cramps in lower limbs, mild cognitive impairment depression, hypotension. | *POLG* NM_002693.2  c.752C>T p.(Thr251Ile) /  c.1760C>T p.(Pro587Leu) in cis  het  WES | ClinVar: Conflicting interpretations of pathogenicity​ (ID 13503 - ID 13505) - these two often occur together  gnomAD NFE genomes: 0.00220 / 0.00220  gnomAD NFE exomes: 0.00263 / 0.00265  Swefreq: 0.0065 / 0.0065  CADD-phred: 24.5 / 13.84  Varsome ACMG: Pathogenic / Pathogenic  Franklin by Genoox: Pathogenic / Likely pathogenic | Possible Spinocerebellar ataxia 28.  Alternative possibility: Digenic interaction of AFG3L2 and SPG7.  The two POLG variants have been described to recur on the same allele in cis and the patient's unaffected daughter carries both POLG variants. Our interpretation is that these variants do not (fully) explain the patient's phenotype.  Although dominant forms of SPG7 have been described, most often this is a recessive disorder.  Heterozygous AFG3L2 variants may cause SCA28, and the p.(Arg702Gln) variant was described in one family with ataxia but with reduced clinical (complete radiological) penetrance [4], supplemental fig.2 in that publication). Our patient reported negative family history. AFG3L2 protein interacts closely with paraplegin, the gene product of SPG7. SCA28 is thus a possible diagnosis, and there might be an interaction of the ARG3L2 and the SPG7 variants, but we found it impossible to make a certain diagnosis. |
|  |  | *AFG3L2* NM_006796.2  c.2105G>A p.(Arg702Gln)  het  WES | ClinVar: Pathogenic-Likely pathogenic​ (ID 5473)  gnomAD NFE genomes: Absent  gnomAD NFE exomes: 0.00000895  Swefreq: Absent  CADD-phred: 32  Varsome ACMG: Pathogenic  Franklin by Genoox: Pathogenic |  |
|  |  | *SPG7* NM_003119.2  c.1454_1462del p.(Arg485_Glu487del)  het  WES | ClinVar: Pathogenic (ID 411680)  gnomAD NFE genomes: 0.000535  gnomAD NFE exomes: 0.000403  Swefreq: Absent  CADD-phred: 32  Varsome ACMG: Pathogenic  Franklin by Genoox: Pathogenic |  |

| P1054  (Fig. 1K) | AO 50yrs. SD 22yrs. Gait, UL & LL ataxia. SARA 13. SARA/SD=0.59/y. Hypometric saccades, saccadic smooth pursuit. MRI/CT: mild CA.  No pyramidal signs.  Areflexia, orthostatic hypotension, reduced sensibility in hands. | *SPAST* NM_014946.4  duplication of 9 exons, chr2:32352018-32372327 (GRCh37/hg19)  het  WES | ClinVar: Identical deletion not reported. Similar deletions of several exons reported as pathogenic.  gnomAD NFE genomes & exomes; Swefreq: NA CADD-phred: NA | Autosomal dominant spastic paraplegia 4 (SPG4) with ataxia?  Patient died as genetic analyses were ongoing. No confirmatory test was performed. Ataxia has been described as a clinical sign of SPG4 in [5], but not without spasticity, as in this patient. We thus interpret the finding as too uncertain. |
| --- | --- | --- | --- | --- |
| P129  (Fig. 1L) | AO 51yrs. SD 8yrs. Gait, UL & LL ataxia. Dysarthria ND. SARA ND. DAT-PET: bilat reduction of dopamine reuptake capacity.  Hyperreflexia.  Polyneuropathy, strabismus, diplopia, cognitive impairment. | *IRF2BPL* NM_024496.4  c.2356G>A p.(Gly786Arg)  het  WES | ClinVar: NR  gnomAD NFE genomes & exomes; Swefreq: Absent  CADD-phred: 31  Varsome ACMG: Likely pathogenic  Franklin by Genoox: VUS | Possibly IRF2BPL-related neurological disorder. Genetic finding appears compatible with our initial clinical data. Renewed clinical examination post NGS is pending. |
| P1051  (Fig. 1M) | AO 61yrs. SD 4yrs. Gait UL ataxia. SARA 5. SARA/SD=1.25/y. MRI/CT: CA and parietal atrophy bilat.  No pyramidal signs.  UL muscle weakness, UL paresthesias, LL impaired vibration sense, LL hyporeflexia. Hypophonia, strabismus. | *SPTBN2* NM_006949.2  c.73C>T p.(Arg25Cys)  het  WGS | ClinVar: Gene not in ClinVar  gnomAD NFE genomes & exomes: Absent  CADD-phred: 22.2  Varsome ACMG: VUS  Franklin by Genoox: VUS  Manual ACMG: VUS (PM2) | Possible Spinocerebellar ataxia 5.  Genetic finding appears compatible with clinical presentation.  Family analyses for co-segregation is ongoing. |
| P1050  (Fig. 1N) | AO 50yrs. SD 8yrs. Gait UL&LL ataxia. Dysarthria. SARA 8. SARA/SD=1.00/y. MRI/CT: normal.  No pyramidal signs.  Diabetes, epileptic seizures, mild unilateral ptosis, muscle cramps, muscle weakness, rigidity, bradykinesia, depression, mild urinary dysfunction. | *RFC1* NM_002913.5  AAGGG (normal: AAAAG)  Repeat lengths estimated to 8/56 but difficult to measure with certainty.  Biallelic  WGS | ClinVar: NR  gnomAD NFE genomes & exomes; Swefreq: NR  CADD-phred: NR | Clinical presentation is not interpreted to be compatible with Cerebellar ataxia, neuropathy, and vestibular areflexia syndrome. |
| P1063  (Fig. 1O) | AO 57yrs. SD 11yrs. Gait, UL & LL ataxia. Dysarthria. SARA 13. SARA/SD=1.18/y. MRI/CT: mild CA and FP atrophy, white matter lesions.  No pyramidal signs.  Diabetes, hyporeflexia, intention tremor, rigidity, nocturia, glaucoma. | *NIPA1* NM_144599.4  deletion exon 1  het  WES | ClinVar: NR  gnomAD NFE genomes & exomes; Swefreq: NA  CADD-phred: NA | No diagnosis established.  Clinical presentation is not interpreted to be compatible with Autosomal dominant spastic paraplegia 6 (SPG6). Deletions are not known causes of SPG6. |
| P1008  (Fig. 1P) | AO 30yrs. SD 8yrs. Gait (w) & UL ataxia. Dysarthria. SARA 9. SARA/SD=1.13/y. GEN, saccadic smooth pursuit, square-wave jerks on fixation, MRI/CT: moderate CA & brainstem atrophy.  Hyperreflexia, spasticity, EPR  Fasciculations in facial muscles, gynecomastia, myopathy, muscle atrophy, distal weakness, myoclonus, hand tremor, weight loss, polyneuropathy. | *POLR3B* NM_018082.6  c.1568T>A p.(Val523Glu)  het  WES | ClinVar: Pathogenic  gnomAD NFE genomes: 0,00086  gnomAD NFE exomes: 0.00060  Swefreq 0.0005  CADD-phred: 24.9  Varsome ACMG: Pathogenic  Franklin by Genoox: Pathogenic  Manual ACMG: Likely pathogenic (PS4 + PM2 (0.06% het but no hom) + PP5) in AR patients. | No diagnosis established.  Although some variants in POLR3B may cause a dominant disease, this particular variant has previously only been described in homozygous or compound heterozygous carriers, causing recessive disease. It is relatively common in population databases. We consider it not to be the cause of the patient's disease. |
| P1086  (Fig. 1Q) | AO 60yrs. SD 5yrs. Gait UL ataxia. SARA 7. SARA/SD=1.40/y. Hypometric saccades, saccadic smooth pursuit, hypoactive VOR. MRI/CT: mild CA, white matter lesions.  Hyperreflexia  Sensorimotor polyneuropathy, tremor left hand and head, depression, impaired vibration sense. | *RFC1* NM_002913.5 AAGGG (normal: AAAAG). Repeat lengths estimated to 56/11 but difficult to measure with certainty.  Biallelic.  WGS.  Confirmed by standard flanking PCR and repeat prime PCR at Genetic Services Laboratory, The University of Chicago, USA. Repeat lengths were not reported. | ClinVar: NR  gnomAD NFE genomes & exomes; Swefreq: NR  CADD-phred: NR | Cerebellar ataxia, neuropathy, and vestibular areflexia syndrome (CANVAS)  There was an AD pattern of inheritance for motor polyneuropathy in the patients’ family, and genes known to cause hereditary polyneuropathy were verified, no genetic cause for polyneuropathy was identified yet.  We hypothesize that this patient may have CANVAS but that both he and his relatives also have a dominant neuropathy for which no genetic cause could be identified. |

**Clinical phenotype and genetic findings in patients where the result is still unclear**

ACMG - American College of Medical Genetics; AO - age at onset; AR - autosomal recessive; CA - cerebellar atrophy; CADD-phred - combined annotation dependent depletion, a tool for scoring the deleteriousness of single nucleotide variants as well as insertion/deletions variants in the human genome; CT - computed tomography; DAT-PET - dopamine transporter imaging with positron emission computed tomography; EPR - extensor plantar response; GEN - gaze-evoked nystagmus; het - heterozygosity; LL - lower limbs; MRI - magnetic resonance imaging; NA – not applicable; ND - not determined; NFE - Non-Finnish-European; NR - not reported; SARA - scale for assessment and rating of ataxia; SD - symptom duration (time from symptom onset to examination within the study); UL - upper limbs; VOR - vestibular-ocular reflex; VUS - variant of uncertain significance; w – wheelchair; WES – whole exome sequencing; WGS – whole genome sequencing.

# Online Resource 3 (text): Additional/more detailed clinical vignettes

**Index patient P1002**, of German extraction, had no family history of ataxia, but an early disease onset at 18 years of age which is suggestive for an autosomal recessive form of ataxia (Fig. 1D). The patient was using a wheelchair when examined by one of the authors and presented clinically with dysarthria, severe ataxia in upper and lower limbs, spasticity, cognitive impairment, ocular apraxia and ophthalmoplegia, hypometric saccades, hypoacusis, dysphagia, muscle atrophy in upper and lower limbs, orthostatic hypotension, and urinary incontinence. Brain MRI showed moderate cerebral and cerebellar atrophy. The genetic testing within this study identified a homozygous variant in the *STUB1* gene, c.761G>A, p. (Arg254His) which previously had been reported as pathogenic. Biallelic pathogenic *STUB1* variants have been associated with autosomal recessive SCAR16 [6]. In this patient both the phenotype and the genotype were characteristic for SCAR16. The patient died at age 42 years due to complications of her severe neurological disease.

**Index patient P1017** belongs to a second family with ataxia-pancytopenia syndrome caused by *SAMD9L* variants. had been referred to the neurology clinic in Lund for investigation of late onset ataxia. Clinically the patient presented with gait and limb ataxia, dysarthria, and horizontal nystagmus. He reported that his mother had a poor balance when she was older, also his brother P1027 and his daughter P1018 presented similar mild symptoms of balance impairment (Fig. 1A). Brain MRI showed moderate cerebellar atrophy. In 2015, within his clinical workup, he was analyzed by Genome Diagnostics Nijmegen with exome sequencing and analyses of ataxia genes known at that time, with negative result. His daughter had had some hematological disturbances when she was a child (Table 2) which in 2017 alerted hematologists and neurologists to the possibility of *SAMD9L* variants, which in 2016 had been described to cause ataxia-pancytopenia syndrome. We asked the genetic laboratory to re-evaluate the data for a variant in this gene. The variant c.2640C>A, p.(His880Gln) was identified that had previously been reported in a large family with ataxia pancytopenia syndrome [7]. This variant was subsequently identified in all the affected family members who were tested. For the detailed clinical phenotype of each member, see Table 2.

**Index patients P1070** and **P1095** are from two different families and developed symptoms at 55 and 60 years of age respectively. Both reported a family history suggestive of an autosomal recessive disease with siblings with similar symptoms. (Fig. 1G/H). The clinical presentation was gait and limb ataxia, impaired vestibulo-ocular reflex, unexplained cough, and sensorimotor polyneuropathy with areflexia, impaired proprioception and vibration sense in the extremities. Brain MRI showed mild cerebellar atrophy for P1070 but no signs of cerebral or cerebellar atrophy for P1095. Analyses of WGS data for repeat expansion within our study revealed altered *RFC1* pentanucleotide composition (AAGGG instead of the normal AAAAG pentanucleotides) but was not able to reliably determine the number of pentanucleotides on each allele (Online Resource 4). Targeted testing using two orthogonal methods at an external laboratory confirmed biallelic extended repeat lengths in the *RFC1* gene, which has been previously described and associated with cerebellar ataxia with neuropathy and vestibular areflexia syndrome (CANVAS) [8]. As a result, we deduced that the phenotype in these two patients is characteristic for CANVAS.

**Patient P1089:** Archimedes spiral drawing at age 63 years (right hand):


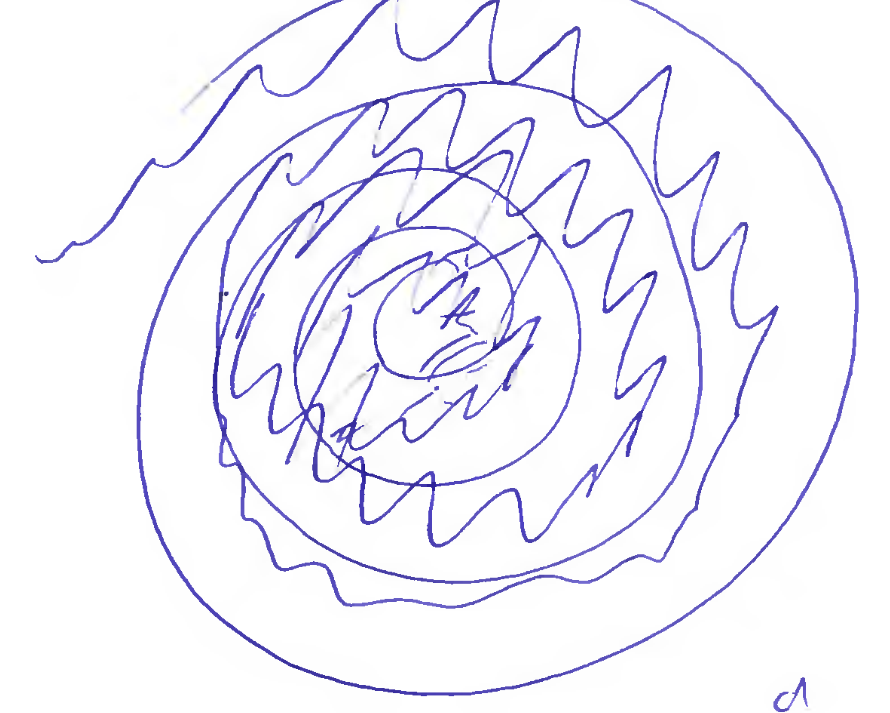


# Online Resource 4 (figure): Analyses of *RFC1* pentanucleotide and *HTT* and *TBP* trinucleotide expansions

Images generated by REViewer v0.2.7 (github.com/Illumina/REViewer), using output from ExpansionHunter v5.0.0 (github.com/Illumina/ ExpansionHunter). The top line shows the human genome reference sequence (with a variable number of repeat units). Horizontal lines below the reference sequence display the single reads obtained by high throughput sequencing. Orange represents repeat sequence, blue represents flanking sequence. Bases are shown as letters in case of mismatch. Horizontal lines represent short deletions, vertical indicate the presence of short insertions. A fainter blue or orange color indicates that this read also could have been assigned to the other allele.

Repeated sequences is an area of genomics ridden with uncertainty, due to the finite read length in “short read” NGS technology. ExpansionHunter and REViewer are not above this limitation, so care must be taken when interpreting these graphs. A guide is available here:

illumina.com/science/genomics-research/articles/reviewer-alignments-short-reads-long-repeat.html.

In brief, if a read is entirely composed of short tandem repeat sequence, one may suspect expansion beyond the estimated repeat unit count (assuming the normal short tandem repeat length is not already longer than the read length). At the other end, if a read fully covers the STR sequence as well as an appreciable amount of flanking sequence, one may conclude that the estimated repeat unit count is accurate.

Long-read NGS technology is advancing rapidly, and its use will markedly improve analyses of short tandem repeats.


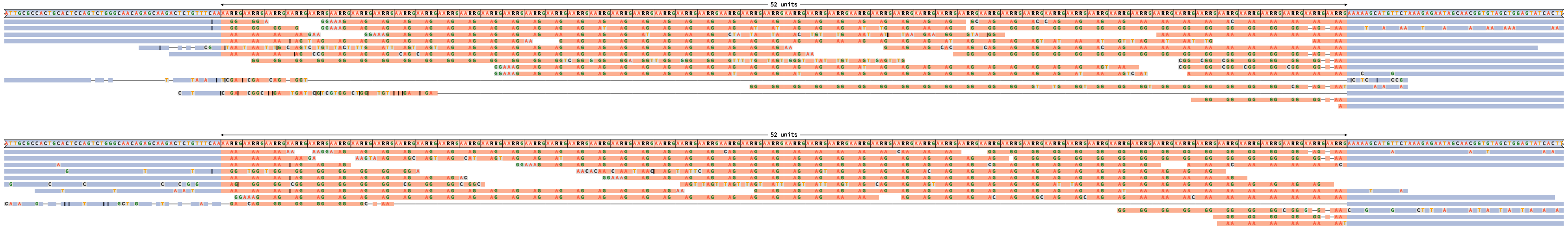


**P1050 (*RFC1* gene pentanucleotide repeat)** The lighter shades indicate homozygosity, as the algorithm could not confidently assign a read to one allele over the other. This sample was not analysed by other methods because the patient’s clinical phenotype was not considered compatible with Cerebellar ataxia, neuropathy, and vestibular areflexia syndrome


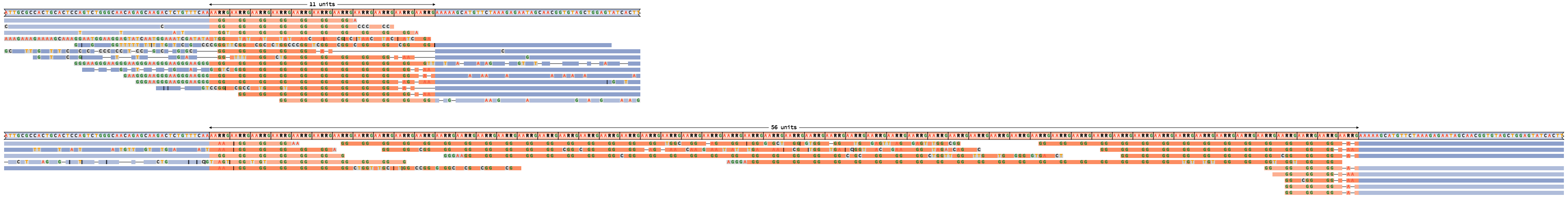


**P1070 (*RFC1* gene pentanucleotide repeat)** After automated alignment, one allele is supposedly not expanded. However, visual inspection reveals that no reads actually support a normal allele. Reads either do not span both ends of the area with pentanucleotide repeats, or do not align well with the reference sequence. Standard flanking PCR and repeat prime PCR at Genetic Services Laboratory, The University of Chicago, USA revealed biallelic pathological repeat expansions; repeat lengths were not reported


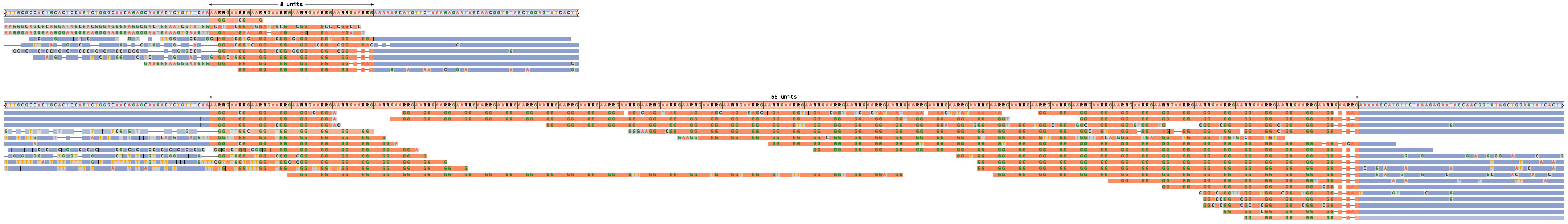


**P1086 (*RFC1* gene pentanucleotide repeat)** The situation is virtually identical to that of P1070. Standard flanking PCR and repeat prime PCR at Genetic Services Laboratory, The University of Chicago, USA revealed biallelic pathological repeat expansions; repeat lengths were not reported


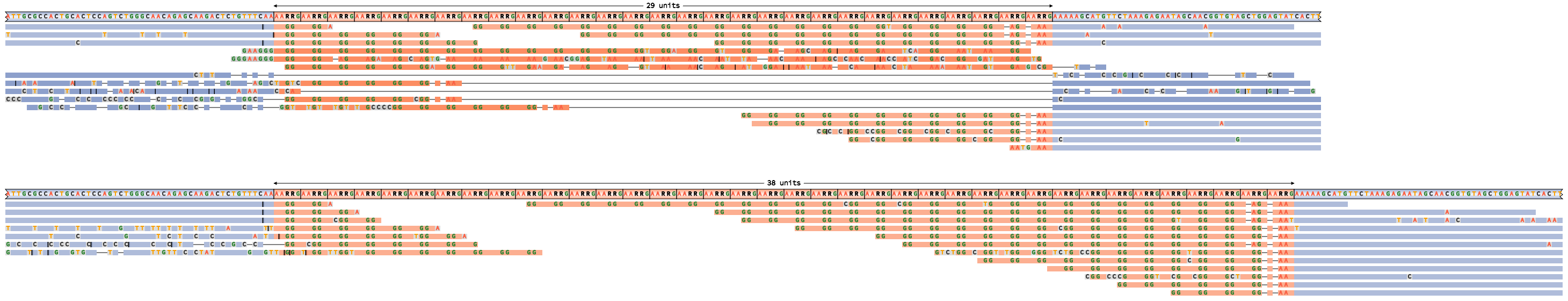


**P1095 (*RFC1* gene pentanucleotide repeat)** As for P1070 and P1086, there is no read in support of a normal allele. Standard flanking PCR and repeat prime PCR at Genetic Services Laboratory, The University of Chicago, USA revealed biallelic pathological repeat expansions; repeat lengths were not reported


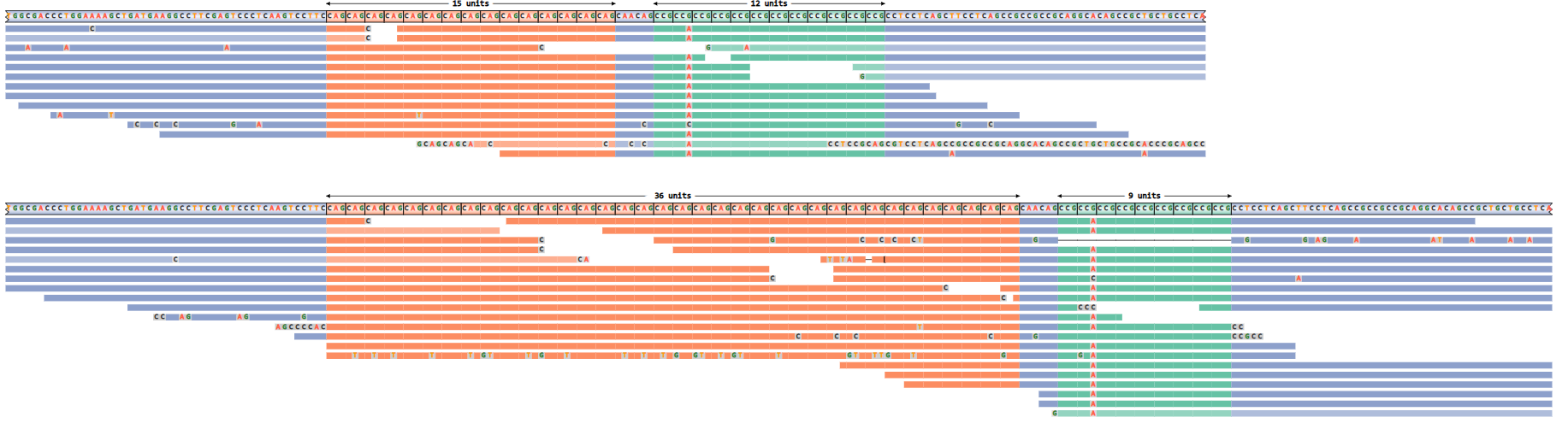


**P1089 (*HTT* gene trinucleotide repeat)** This sample shows 36 consecutive CAG repeats on one allele of the HTT gene and 15 repeats on the other allele followed by CAA-CAG (CAA interruption) and by CCG repeats (green). Though the CAG repeat is expanded, its total length, in terms of number of bases, is appreciably shorter than the read length; several reads span all the CAG units as well as both flanking regions. Thus, the repeat unit number estimation, 36, is not ambiguous here unlike for the RFC1 cases above.


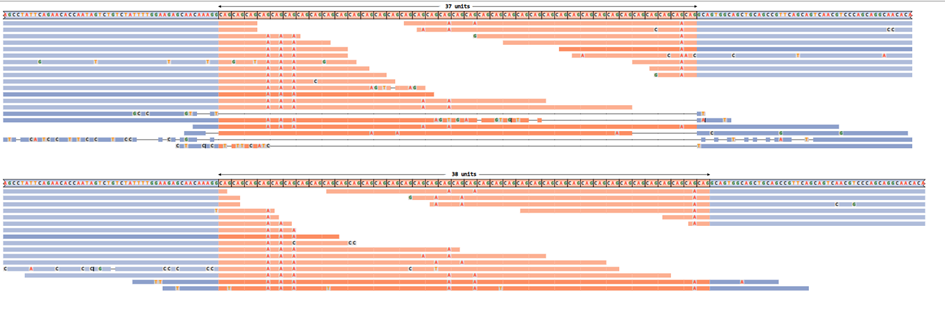
**P1040 (normal length of TBP repeat)** in this patient with SCA48.

# References to Online Resources

1. Zerr, P., J.P. Adelman, and J. Maylie, *Episodic ataxia mutations in Kv1.1 alter potassium channel function by dominant negative effects or haploinsufficiency.* J Neurosci, 1998. **18**(8): p. 2842-8.

2. Hadjivassiliou, M., et al., *Causes of progressive cerebellar ataxia: prospective evaluation of 1500 patients.* Journal of Neurology, Neurosurgery &amp; Psychiatry, 2017. **88**(4): p. 301-309.

3. Pandolfo, M. and M. Manto, *Cerebellar and afferent ataxias.* Continuum (Minneap Minn), 2013. **19**(5 Movement Disorders): p. 1312-43.

4. Gorcenco, S., et al., *New generation genetic testing entering the clinic.* Parkinsonism Relat Disord, 2020. **73**: p. 72-84.

5. Coutinho, P., et al., *Hereditary ataxia and spastic paraplegia in Portugal: a population-based prevalence study.* JAMA Neurol, 2013. **70**(6): p. 746-55.

6. Ravel, J.-M., et al., *Expanding the clinical spectrum of STIP1 homology and U-box containing protein 1-associated ataxia.* Journal of Neurology, 2021. **268**(5): p. 1927-1937.

7. Chen, D.H., et al., *Ataxia-Pancytopenia Syndrome Is Caused by Missense Mutations in SAMD9L.* Am J Hum Genet, 2016. **98**(6): p. 1146-1158.

8. Cortese, A., et al., *Biallelic expansion of an intronic repeat in RFC1 is a common cause of late-onset ataxia.* Nat Genet, 2019. **51**(4): p. 649-658.
